# Intrathecal pump refills at home or at the hospital: Protocol for a randomized controlled crossover trial—The IMPROVE study

**DOI:** 10.1371/journal.pone.0354092

**Published:** 2026-07-27

**Authors:** Ulrike Van Hoey, Britt Winnepenninckx, Maarten Moens, Koen Putman, Lisa Goudman

**Affiliations:** 1 STIMULUS Research Group, Vrije Universiteit Brussel, Jette, Belgium; 2 Department of Neurosurgery, Universitair Ziekenhuis Brussel, Jette, Belgium; 3 Center for Neurosciences (C4N), Vrije Universiteit Brussel, Jette, Belgium; 4 Pain in Motion Research Group (PAIN), Department of Physiotherapy, Human Physiology and Anatomy, Faculty of Physical Education & Physiotherapy, Vrije Universiteit Brussel, Jette, Belgium; 5 Research Foundation Flanders (FWO), Brussels, Belgium; 6 Department of Radiology, Universitair Ziekenhuis Brussel, Jette, Belgium; 7 Interuniversity Centre for Health Economics Research (I‑CHER), Department of Public Health (GEWE), Faculty of Medicine and Pharmacy, Vrije Universiteit Brussel, Jette, Belgium; UCSI University, MALAYSIA

## Abstract

**Background:**

Intrathecal drug delivery (IDD) offers a therapeutic option for patients suffering from refractory pain or severe spasticity. By allowing targeted and continuous infusion directly into the intrathecal space, IDD bypasses the blood-brain barrier and enhances therapeutic effectiveness of the drug. Following the implantation of an IDD pump, the most commonly performed postoperative maintenance procedure is the pump refill (at regular intervals). This process can be burdensome for patients, affects their comfort, and carries significant risks. The current aim of this study is to evaluate whether intrathecal pump refills performed at home provide a difference in patient comfort compared to refills conducted in the hospital.

**Methods:**

The IMPROVE study is a monocentric, randomized controlled crossover trial, including 82 patients. For this study, each patient will undergo four intrathecal pump refill procedures (two at home and two in the outpatient clinic) allocated in a randomized order. The primary objective of this study is to determine whether at-home refills provide a difference in patient comfort compared to hospital-based refills. Secondary objectives include assessing differences in quality of life, pain, stress, anxiety, self-efficacy, caregiver burden, patient preferences, safety, and overall cost-effectiveness between the two settings. Patients will be followed over the course of four intrathecal pump refills, which is estimated to span approximately one year.

**Discussion:**

Within the IMPROVE project, pump refills will be performed through hospital at home. If at-home intrathecal pump refills prove more comfortable for patients and cost-effective for society, this would strengthen the patient-centred care model and support adopting this approach as the new standard treatment for IDD patients. A graphical abstract is provided in the supplementary materials (S1 Fig).

**Trial registration:**

Details on the study site can be found at ISRCTN with identifier: ISRCTN18031921; [href:https://doi.org/10.1186/ISRCTN18031921]https://doi.org/10.1186/ISRCTN18031921. The trial was registered in the ISRCTN registry on 18 November 2025.

## Introduction

Intrathecal analgesia offers an effective therapeutic option for patients with refractory pain or those unable to tolerate the adverse effects of conventional treatments. It is often regarded as a final-line intervention, reserved for individuals who have suffered from chronic, long-standing pain (typically over several years) and have not achieved adequate analgesia despite escalating doses of systemic opioid therapy [1]. Consequently, intrathecal analgesia may be considered for the management of non-oncologic pain in patients with conditions such as compression fractures, spondylolisthesis, spondylosis, persistent spinal pain syndrome type II, and spinal stenosis [2].

Beyond pain management, intrathecal drug delivery (IDD) also plays a vital role in the treatment of severe spasticity [3], particularly in patients who do not respond adequately to oral antispastic agents or experience intolerable side effects. Intrathecal administration of agents such as baclofen allows for direct delivery to the cerebrospinal fluid, resulting in improved symptom control at significantly lower doses and with fewer systemic effects [4,5].

**Fig 1:**
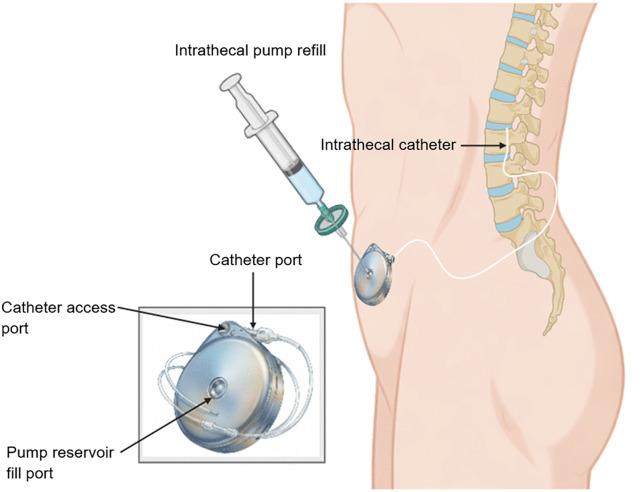
Schematic representation of an intrathecal drug delivery (IDD) system and refill procedure. The pump is implanted subcutaneously in the abdominal region and connected to an intrathecal catheter, which delivers medication directly into the cerebrospinal fluid. The pump contains a pump reservoir fill port, catheter access port and catheter port. The intrathecal pump refill is performed by percutaneously inserting a needle through the skin into the reservoir fill port, allowing aseptic replacement of medication.

IDD provides targeted and continuous infusion into the intrathecal space through a pump implanted subcutaneously in the abdominal region. This pump is connected to a catheter that delivers medication directly into the cerebrospinal fluid (Fig 1), thereby effectively bypassing the blood-brain barrier (BBB) [2]. One of the greatest challenges in delivering pharmacological treatments to the central nervous system is the restrictive nature of the BBB, which delays nearly 98% of systemically administered medications from reaching the cerebrospinal fluid [2,6]. By administering drugs within the intrathecal space, far lower dosages can be used compared to systemic routes. As a result, systemic exposure to medication remains minimal or even undetectable, potentially lowering the risk of adverse effects [2,7].

Following the implantation of an IDD pump, the most commonly performed postoperative maintenance procedure is the drug refill. This involves aseptic access to the pump’s reservoir fill port, removal of any remaining medication, and filling the reservoir with new medication [8] (Fig 1). A step-by-step refill protocol is provided in S2 Fig.

Successful therapy requires ongoing follow-up by specialists, as pump refills must be carried out at regular intervals in an outpatient hospital setting [9]. This process can be burdensome for patients and carries significant risks. Delayed refills may lead to severe withdrawal symptoms, and in the case of baclofen, potentially life-threatening complications such as rhabdomyolysis, seizures, coma, or even death [10]. Consequently, intrathecal pump refills and intrathecal pump malfunctioning are allocated as urgent patient procedures [11].

Within our previous work, we demonstrated that intrathecal pump refills can be safely and successfully performed in a hospital at home (HAH) setting using telemonitoring support [12]. These findings align with those from a retrospective analysis conducted in the Netherlands, which also reported positive outcomes for on-site refill procedures as part of routine pump aftercare [4,13]. By performing refills through HAH, the burden, stress, and increased pain associated with hospital travel for both patients and informal caregivers can be avoided [4]. Despite these benefits of HAH, a head-to-head comparison of refill procedures at the hospital or at home and their cost effectiveness has yet to be conducted. With IMPROVE, we want to nationally deliver an intervention that not only increases patient comfort but also proves to be cost effective for society.

## Materials and Methods

### Objectives

The primary objective of this study is to assess whether intrathecal pump refills conducted at home provide a difference in patient comfort compared to hospital-based refills. Additionally, the study aims to explore potential differences between home- and hospital refills regarding health-related quality of life (HRQoL), pain intensity, pain interference, stress, anxiety, general self-efficacy, caregiver burden, patient preferences concerning the refill location, and the occurrence of (serious) adverse events ((S)AEs). A final objective is to conduct an economic evaluation comparing home-based and hospital-based intrathecal pump refills, considering healthcare expenditures as well as direct and indirect costs.

### Patient and public involvement

This study was initiated in response to patient feedback indicating that frequent hospital visits for intrathecal pump refills were burdensome and placed additional strain on their caregivers. The research question was therefore patient-driven and subsequently developed in collaboration with clinicians. The feasibility of the proposed approach was evaluated in a pilot study and found to be acceptable to both patients and physicians [12].

### Trial design

**Fig 2:**
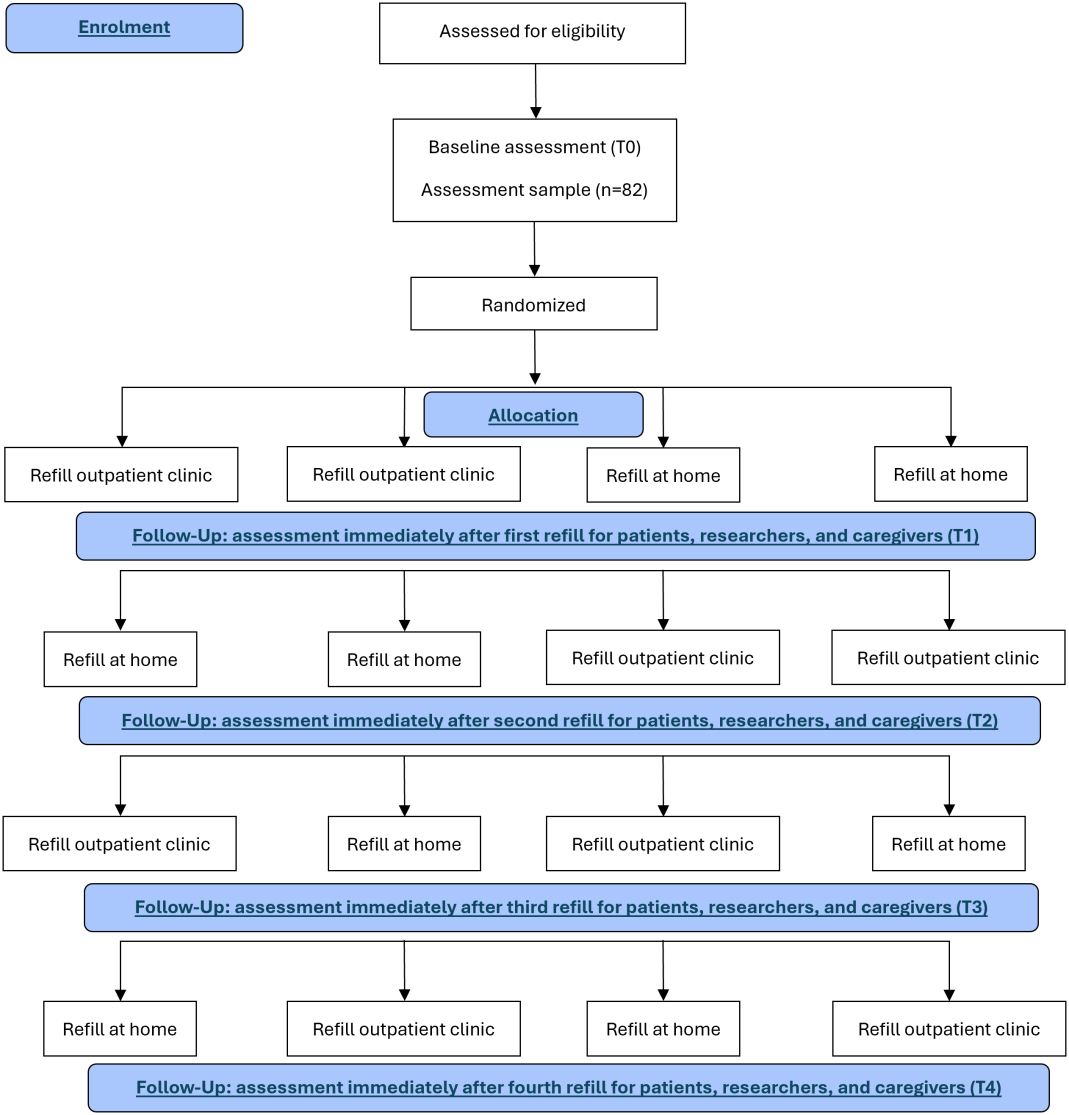
Flowchart of the IMPROVE-study. Each patient will receive two refills at the outpatient clinic and two refills at home, in a randomized order. The refills are grouped in pairs, with each pair consisting of one home refill and one hospital refill.

IMPROVE is a monocentric, randomized controlled crossover trial designed to assess whether intrathecal pump refills conducted at home offer differences in patient comfort compared to refills performed in a hospital setting. Each participant will undergo four refill procedures (two at home and two in the outpatient clinic) allocated in a randomized order (Fig 2).

### Trial setting

The IMPROVE study will be conducted in University Hospital Brussels, located in Belgium. The study protocol (version 3.0) was approved by the Ethics Committee (EC) of the Universitair Ziekenhuis Brussel/Vrije Universiteit Brussel on 3 December 2025. Written informed consent will be obtained from all participants prior to participation. In the future, at least one additional study site will be included in the protocol to ensure the required sample size is achieved.

### Eligibility criteria

In this study, patients with chronic non-cancer pain or spasticity who receive IDD are eligible to participate.

Inclusion criteria are:

- Adult patients, 18 years or older
- Actively receiving IDD
- Stable medication dosage for at least three months
- Dutch, French or English speaking

Exclusion criteria are:

- Life-expectancy less than six months
- Patients planned for, but not yet received IDD implant
- No residence in Belgium

### Consent or assent

#### Who will obtain informed consent?.

Eligible patients will first be informed about the project by their treating physician. Subsequently, a researcher from the IMPROVE consortium will be notified and will contact the patient to provide additional information. The patient will then be screened according to the inclusion and exclusion criteria. Patients who remain eligible and express continued interest in participating will receive detailed oral and written information about the study and will have the opportunity to ask questions. Written informed consent will be obtained prior to participation.

#### Additional consent provisions for collection and use of participant data and biological specimens.

For the collection of saliva samples using synthetic salivettes (Sarstedt AG & Co., Nümbrecht, Germany), consent will be obtained as part of the informed consent process.

### Intervention and comparator

With IMPROVE, we propose to perform intrathecal pump refills at the patient’s home instead of at the hospital, in a population of patients with chronic pain or severely disabling spasticity.

**Fig 3:**
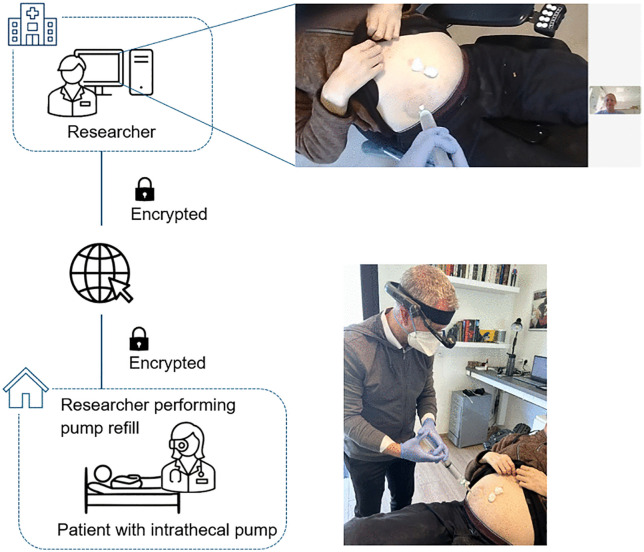
Architecture of remote monitoring system. A remotely located researcher in the hospital provides real‑time supervision via an encrypted connection through the RealWear Navigator 520 to the researcher performing the intrathecal pump refill at home.

The experimental intervention consists of two pump refills per patient, with the refill procedure and protocol remaining identical to the current standard of care used in the hospital (S2 Fig). However, differences exist between the home- and hospital-based procedure settings (S3 Fig). Instead of receiving the refill at the hospital, it will be conducted in the patient’s home environment. A researcher will visit the patient and carry out the procedure under remote control using smart glasses (RealWear Navigator 520). This enables real-time communication between the researcher performing the home-based refill and the remotely located hospital-based researcher. The RealWear Navigator 520 is General Data Protection Regulation (GDPR)-compliant and supports both Bluetooth and Wi-Fi connectivity, allowing connection to an external internet source. A secure end-to-end connection is established via Microsoft Teams, with live transmission only; no visual or audio data are recorded or stored (Fig 3). A checklist for home-based intrathecal pump refills covering pre-visit preparation, required materials, home environment setup, the refill procedure, post-procedure care, and follow-up is provided in S5 Fig.

**Fig 4:**
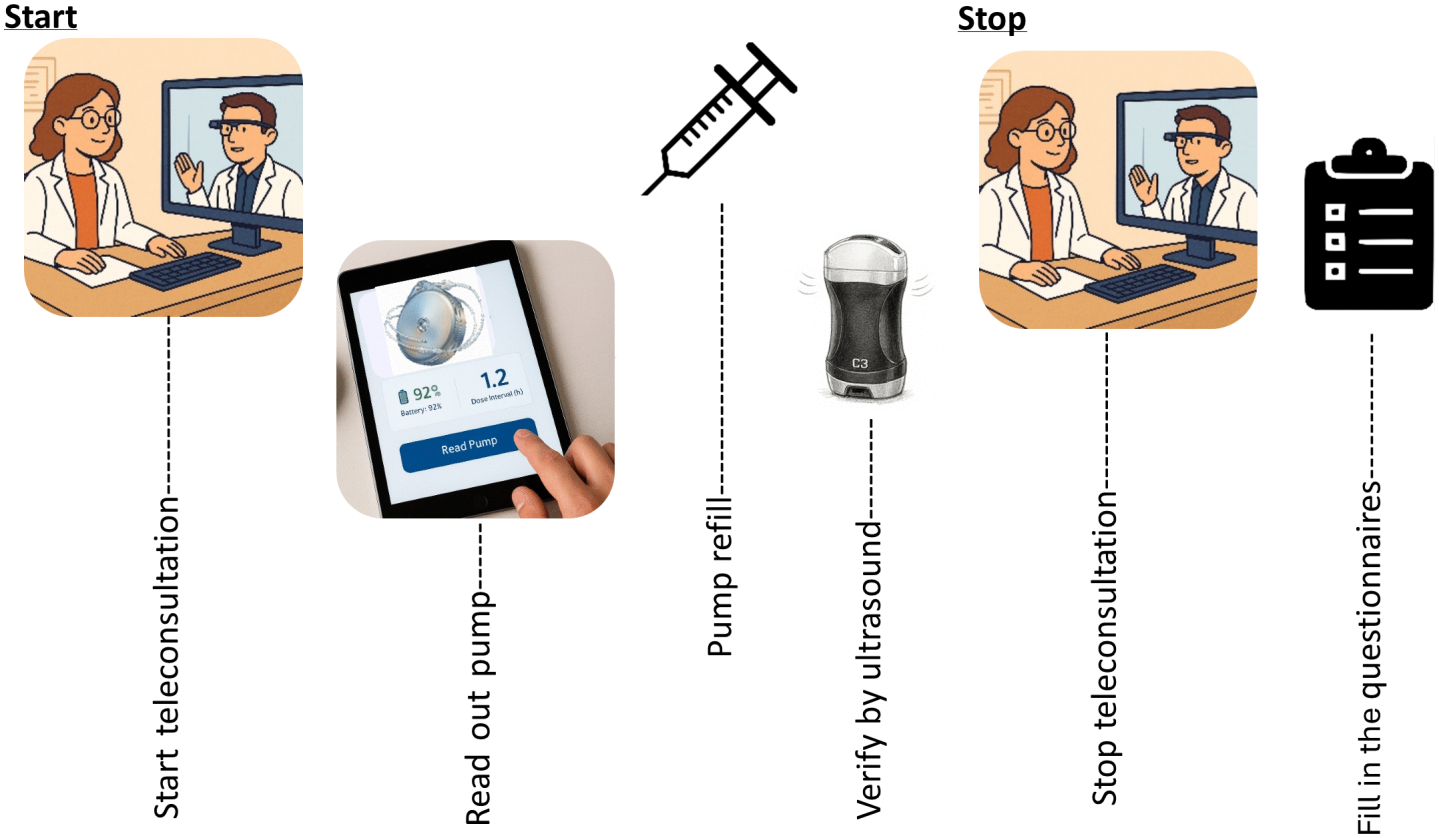
Overview intrathecal pump refill at home. Teleconsultation is set up by the researcher at the patient’s home, followed by reading out the intrathecal pump, performing the pump refill procedure, ending the teleconsultation, and completion of the questionnaires after the procedure by the participants.

Following the refill, a post-refill evaluation using an ultrasound device (Clarius C3 Scanner, Vancouver, Canada) will be performed to confirm that the pump was correctly refilled and that no subcutaneous drug injection occurred (Fig 4). Follow-up will be conducted by phone 2-12 hours after the pump refill to assess any AEs.

If the procedure cannot be carried out in a clean and sterile manner at the patient’s home (e.g., due to poor hygiene or inadequate general body care), the home refill attempt will be considered unsuccessful. In that case, the procedure will be rescheduled and performed at the outpatient clinic within the same week. In the control group, standard care is provided: the patient travels to the hospital where the physician or nurse refills the pump.

### Criteria for discontinuing or modifying allocated interventions

The participants can withdraw from the study at any time.

### Strategies to improve adherence to interventions

At each refill, an appointment will be scheduled for the next one. To support participant retention and ensure completion of study procedures, patients will receive a reminder of their upcoming refill appointment a couple of days before. Following each refill, they will be given a tablet with the relevant questionnaires, in the presence of the researcher.

Fidelity, defined as the extent to which delivery of an intervention adheres to the protocol originally developed [14], will be monitored in two ways: for home-based refills, a researcher at the hospital will observe the procedure remotely via telemonitoring; for hospital-based refills, a researcher will conduct occasional on-site checks to ensure protocol adherence.

### Relevant concomitant care permitted or prohibited during the trial

There are no prohibitions during the trial.

### Outcomes

During the baseline assessment, patients are asked to provide information on their demographic and socio-economic background as well as clinical baseline data. Further, patients will receive questionnaires related to our primary and secondary outcome measures (Table 1), an Electronic Data Capture system “REDCap (Research electronic Data Capture)” will be used for data collection.

**Table 1:** Overview of outcomes of the IMPROVE study.

What?	Patient comfort *	QoL *	Pain Intensity*	Pain interference *	Stress *	Anxiety *	Self-efficacy*	Burden caregivers*	Patient satisfaction	Tele-monitoring quality** + time	Safety	Health expenditure ***	Patient preference
How?	GCQ	MQOL+ EQ-5D-5L	VAS	PROMIS	Sarstedt AG & Co	STAI	GSE	ZBI	Likert-scales	Likert-scales + time expenditure	Overall safety + environmental safety + sterile procedure	Phone calls	Multiple choice + DCE
When?	At baseline * and after each intrathecal pump refill (** only applicable for refills at home)***Four weeks after first refill and four weeks after second refill												After last refill
Who?	Patient	Patient	Patient	Patient	Salivary sample	Patient	Patient	Caregiver	Patient	Patient + researchers	Patient + researchers	Patient	Patient

Abbreviations: GCQ = General Comfort Questionnaire; MQOL = McGill Quality of Life Questionnaire; MQOL+ EQ-5D-5L = EuroQol with five dimensions and five levels; VAS = Visual Analogue Scale; PROMIS = Patient-Reported Outcomes Measurement Information System; STAI = State Trait Anxiety Inventory; GSE = General Self-Efficacy; ZBI = Zarit Burden Interview, DCE = Discrete Choice Experiment.

After each refill, all baseline outcome measures will be reassessed. In addition, secondary outcomes such as patient satisfaction and preference, telemonitoring quality (applicable only for home refills), and safety will be evaluated during follow-up assessments. Health expenditure data will be collected through phone calls. The researchers will call each patient four weeks after the first refill and four weeks after the second refill. To prevent test order effects, the order of the self-reported measures will be randomized for each individual patient at each assessment. After the fourth refill patients will be asked to complete a Discrete Choice Experiment (DCE).

### Primary outcome

The General Comfort Questionnaire (GCQ) will serve as the primary outcome measure, enabling the evaluation of perceived comfort across both refill settings. This concept is grounded in Kolcaba’s mid-range theory of comfort, which defines comfort as “The immediate experience of being strengthened by having needs for relief, ease, and transcendence met in four contexts: physical, psychospiritual, sociocultural, and environmental, and it is so much more than the absence of pain” [15,16]. GCQ is a self-reported instrument consisting of 48 items that reflect the physical, spiritual, environmental and social dimension [15]. Responses are rated on a four-point Likert scale ranging from “strongly disagree” to “strongly agree”. Some items are reverse scored during data processing, with higher overall scores indicating greater levels of comfort [17].

### Secondary outcomes

**Health-related Quality of Life.** The McGill Quality of Life Questionnaire (MQOL) will be used to describe the QoL. This questionnaire consists of 17 questions. Sixteen of these are rated on an 11-point Likert scale (range from 0 to 10) and assess various domains including physical symptoms, physical wellbeing, psychological symptoms, perception towards life and sense of achievement [18,19]. The final part is an open question. Higher scores reflect a better QoL [18,19]. Originally this questionnaire was developed for palliative patients [19], the MQOL has since been validated for patients with chronic conditions [18]. It captures a multidimensional view of QoL by evaluating physical wellbeing, psychological wellbeing, existential wellbeing, social support, and physical symptoms, along with an overall QoL rating [18].

Additionally, the EuroQol with five dimensions and five levels (EQ-5D-5L) will be assessed to describe the health-related QoL. According to the Belgian EQ-5D-5L value set by Bouckaert et al. (2022), index scores range from −0.42 to 1. A score of 0 represents death, while a score of 1 indicates perfect health. These values are based on preferences from the Belgian population [20]. Patients subjectively select the statement that best describes their status within each of the five dimensions: mobility, self-care, usual activities, pain/discomfort, and anxiety/depression [21].

**Pain intensity.** The Visual Analogue Scale (VAS – 100 mm) in electronic format will be used for the assessment of current pain intensity. The VAS pain score is reliable, valid, and sensitive to change [22,23]. VAS measurements are found to have good test-retest reliability [24].

**Pain interference.** Pain interference refers to the degree to which pain hinders or restricts an individual’s physical, emotional, and social functioning [25]. To measure this impact systematically, the Patient-Reported Outcomes Measurement Information System (PROMIS), developed and supported by the National Institutes of Health (NIH), offers standardized, reliable tools for assessing clinical outcomes across diverse patient populations [26]. The PROMIS pain interference measure specifically captures how pain affects participation in everyday activities, including social, emotional, cognitive, physical, and recreational domains. It has demonstrated strong psychometric properties, including reliability and construct validity, supporting its use as an outcome measure in chronic pain settings [25].

**Stress.** Saliva samples will be collected to measure cortisol levels. This provides a non-invasive, valuable and objective method for assessing Hypothalamo-Pituitary-Gland (HPA) axis activity and stress levels [27]. These samples will be obtained with synthetic salivettes (Sarstedt AG & Co., Nümbrecht, Germany) 5 minutes before the start of the refill procedure, immediately after the refill procedure and 10 minutes afterwards. Saliva samples will be stored at −20°C until analysis. The saliva samples will be analysed using the Cobas 8000: Elecsys® Cortisol II, electrochemiluminescence immunoassay.

**Patient anxiety.** Patient anxiety will be provided through the State Trait Anxiety Inventory (STAI). The STAI, developed by Spielberger, Gorsuch, and Lushene, is designed to provide an objective assessment of anxiety levels in psychologically healthy adults [28]. This questionnaire consists of 40 Likert-type items on a four-point scale. STAI is widely used in chronic pain research to assess anxiety, as it captures both acute anxiety symptoms and anxiety as a stable trait of the patient’s personality [28,29]. For both types of anxiety, the total score ranges from 20 to 80, with higher scores indicating higher levels of anxiety. The STAI is a highly reliable measure that can discriminate between high- and low-stress situations [28].

**Self-efficacy.** The General Self-Efficacy (GSE) Scale is a 10-item self-report questionnaire used to assess perceived self-efficacy. This construct reflects an individual’s belief in their ability to cope with challenging situations and to successfully achieve goals [30,31]. In the context of chronic pain, it encompasses not only the expectation of being able to perform specific tasks or behaviors, but also the confidence to do so despite the presence of pain [31–33]. Higher self-efficacy scores have shown to be associated with lower pain intensity levels, better physical health-related QoL, better mental health related QoL, lower catastrophizing, and use of coping strategies [32,33]. The GSE Scale has been used in chronic pain conditions [34]. Its validity was assessed in participants dealing with stressful health-related situations [35].

**Caregiver burden.** To evaluate caregiver burden, the Zarit Burden Interview (ZBI) questionnaire will be completed [36]. This 12-item self-report instrument assesses caregiver burden and is a shortened version of the original 22-item Zarit Burden Scale [37–39]. Each item is rated on a 5-point scale, with higher scores indicating greater burden; a score of 4 on any item reflects the highest level of perceived strain. The total score ranges from 0 to 48, with higher totals reflecting more significant caregiver burden. The questionnaire focuses on aspects such as time demands, physical health, mental strain, and psychosocial stressors [38].

**Patient satisfaction.** Patient satisfaction is evaluated with a seven-point Likert scale asking the patient to rate the overall level of satisfaction with the refill at home.

**Tele-monitoring quality.** The quality of tele-monitoring will only be evaluated after a home-filling by the researcher at home and the researcher in the hospital. This will be examined using three different Likert scales to score 1) quality of audio, 2) quality of video, and 3) overall quality of the teleconsultation [40].

**Time of the refill.** The time required for the procedure will be recorded from three perspectives. First, the time for the refiller will be measured: for home-based refills, this includes the duration from entering the patient’s house until departure. For hospital-based refills, it covers the time spent in the patient’s room in the outpatient clinic. Second, the time required for medical supervision will be recorded: for home-based refills, this includes the full duration of the online consultation. For hospital-based refills, it includes the time the physician spends in the outpatient clinic with the patient. Thirdly, the time spent by the patient will be measured. For home refills, the time recorded will be the same as that of the refiller. For hospital refills, the recorded time will include the entire duration from the patient’s departure from home prior to the refill until their return home afterwards.

**Safety.** Patients will evaluate the overall safety of the procedure using a seven-point Likert scale. In addition, both the researchers (at home and at the hospital) will assess safety on three levels: (1) overall perceived safety of the procedure using a seven-point Likert scale; (2) environmental safety, assessed through an open-ended question addressing any situations perceived as unsafe; and (3) the ability to perform the procedure in a clean and sterile manner, also evaluated via an open-ended question. Between two and twelve hours after the refill at home, the researcher at the hospital will contact all patients by phone to assess the occurrence of any AEs.

**Health expenditures.** Expenditures related to in-hospital care will be extracted from hospital claims data. All other healthcare-related costs will be gathered through telephone interviews with patients, conducted four weeks after the first and second medication refills. During these calls, researchers will ask patients whether they have had any medical consultations, hospital admissions, AEs, changes in medication, or incurred any additional healthcare costs.

**Patient preference.** After the fourth refill procedure, patients will be asked to indicate their preference (refill at home, refill in the hospital, no preference or other) for the location of their refills in the future. Additionally, patients will be presented a DCE to evaluate their preferences across several scenarios to conduct a willingness-to-pay analysis.

To improve clarity, a comparative overview of expected clinical, logistical, and patient-centered aspects of home-based versus hospital-based refill procedures is provided in S1 Table.

### Adverse events reporting and harms

#### Documentation and reporting of adverse events.

All AEs and adverse reactions (ARs), whether spontaneously reported by the patient or observed by the assessor, will be documented from baseline until the end of the study. For the purpose of safety evaluation, refill-associated infections will include, but are not limited to, pocket infections, catheter tract infections, meningitis, or infections requiring antibiotic treatment or hospitalization. The relationship of any reported (S)AE to the refill procedure will be carefully assessed by the steering committee and classified as definitely, probably, unlikely, or not related. All SAEs will be reported to the Competent Authorities (CA) and EC within 24 hours. All suspected unexpected serious adverse events (SUSARs) must be reported to the EC and health authority as per European Union (EU) and Belgian legislation. The reporting period for SUSARs, if fatal or life-threatening, is as soon but not later than 7 days after becoming aware. For SUSARs, not fatal or non-life threatening, the reporting period is 15 days after becoming aware. The sponsor will report to the EC and Health Authorities.

#### Overdose and pocket refill management.

Home-based refills were introduced to reduce the burden on patients and improve access to care, while maintaining safety standards equivalent to hospital-based procedures through structured training, standardized protocols, and post-refill verification. Because a home-based intrathecal pump refill is an invasive procedure, dedicated safety measures are implemented to minimize potential risks. Refills are performed by experienced clinicians using a standardized refill protocol and aseptic technique. Following each refill, ultrasound verification (Clarius C3 Scanner, Vancouver, Canada) is performed, to confirm that the pump was correctly refilled and that no subcutaneous drug injection occurred.

In case of suspected pocket refill or symptoms suggestive of overdose, emergency medical services (112) are contacted immediately while the healthcare professional remains with the patient and initiates supportive measures until transfer to emergency care. An emergency flowchart is illustrated in S4 Fig.

Morphine overdose can manifest as respiratory depression, decreased consciousness, seizures, or bradycardia. Intrathecal baclofen overdose may present with hypotonia, decreased consciousness or coma, respiratory depression, hypotension, nausea/vomiting, or seizures. Until transfer to emergency care is provided, supportive measures including airway management and patient positioning are initiated.

### Participant timeline

The participant timeline for the IMRPOVE study is presented in Table 2. Exact timings cannot be provided, as they depend on the patient’s daily dose and the volume of the pump.

**Table 2:** Timeline participants for the IMPROVE study.

	Study period					
	Enrolment	Allocation	Post-allocation			
Timepoint	Baseline (T0)	0	After first refill (T1)	After second refill (T2)	After third refill (T3)	After fourth refill (T4)
**ENROLMENT:**						
Eligibility screen	X					
Informed consent	X					
Demographic data	X					
Randomization Allocation		X				
**ASSESSMENTS:**						
GCQ, MQOL, EQ-5D-5L, VAS, PROMIS, saliva samples, STAI, GSE, ZBI, *Health-economics telephone interview	X		X (* + 4 weeks)	X (* + 4 weeks)	X	X
**Patient satisfaction, telemonitoring quality, time, safety			X	X	X	X
DCE						X
Preference						X

Exact timings cannot be provided, as they depend on the patient’s daily dose and the volume of the pump. Abbreviations: GCQ = General Comfort Questionnaire; MQOL = McGill Quality of Life Questionnaire; EQ-5D-5L = EuroQol with five dimensions and five levels; VAS = Visual Analogue Scale; PROMIS = Patient-Reported Outcomes Measurement Information System; STAI = State Trait Anxiety Inventory; GSE = General Self-Efficacy; ZBI = Zarit Burden Interview; DCE = Discrete Choise Experiment. **Only applicable for refills at home.

### Sample size

Sample size estimation, conducted using G*Power version 3.1.9.4, was based on patient comfort (assessed via the GCQ) as the primary efficacy outcome variable following four intrathecal pump refill procedures. Estimated true mean comfort scores were derived from the study by Zhao et al. (2021) [41], while a pooled standard deviation was calculated from three relevant studies [41–43], resulting in a value of 16.01049. Based on these parameters, the effect size (f) was determined to be 0.3035623. For interpretability, this corresponds approximately to a Cohen’s d of 0.60, which represents a medium-sized effect according to conventional benchmarks described by Cohen.

The study is designed as a randomized clinical trial in which each patient undergoes four pump refill procedures: two at home and two in the outpatient clinic. The expected mean comfort scores are 88.78 for home-based refills and 78.47 for clinic-based refills. Using the estimated common standard deviation of 16.01049, a total sample size of 82 patients is required to detect a statistically significant difference between the two settings, assuming a two-sided alpha level of 0.05 and a power of 85%. This sample size considers a 20% loss to follow-up after four refill procedures.

### Recruitment

Patient recruitment will take place at University Hospital Brussels (Belgium). Patients have been recruited from October 2025 onwards. The recruitment phase is anticipated to last 28 months, targeting an average inclusion rate of three patients per month. Data collection is expected to be completed approximately one year after the end of recruitment, with results anticipated six months thereafter. However, enrolment will persist until the required sample size is achieved, irrespective of the recruitment timeline. If patient inclusion progresses slowly, additional hospitals may be contacted to support recruitment. Eligible patients will be informed about the study by neurosurgeons, anaesthesiologists, nurses, or other healthcare professionals during scheduled refill appointments.

### Randomization

#### Sequence generation.

Each participant will undergo four refill procedures: two at home and two in the outpatient clinic. The order of these procedures will be randomized, with the allocation sequence generated in R by the researcher team. To counter time-related confounding effects on the outcome measures, the procedures are organized into paired sessions, with each pair consisting of one home-based and one hospital-based refill. This design results in four distinct randomization pathways (Fig 2). Restricted randomization will be conducted during the week following the baseline assessment.

Given the short interval between the paired procedures and the within-subject crossover design, the potential period and sequence effects are expected to be minimal. Therefore, the primary analysis will focus on within-subject comparisons between the two settings.

#### Allocation concealment mechanism.

To ensure allocation concealment, the randomization sequence was generated in advance and stored in a secure, access-restricted Excel log. Group assignments are only revealed after participant enrolment and baseline data collection.

#### Implementation.

The researcher is responsible for informing patients of their assigned treatment track. Furthermore, the researcher will communicate which refill procedures will take place at the hospital and which will be conducted at home. An exact date will be provided only for the first refill. For subsequent refills, an appointment will be scheduled during each refill visit.

#### Blinding.

The statistician will be blinded to group allocation. Blinding will be achieved by storing the collected data in an access-restricted Excel log. Patients and the researchers performing the refills cannot be blinded to the refill location. To minimize bias, patients will complete the study outcome measures electronically, ensuring that the outcome assessor cannot influence their responses.

#### Unblinding.

As blinding applies only to the statistician, there are no circumstances under which unblinding would be permissible during the trial. No procedure for revealing a participant’s allocated intervention is foreseen.

### Data collection

#### Plans for assessment and collection of trial data.

The self-reported measures will be completed online via REDCap to enhance study feasibility and ensure data security. For participants who are unable to complete the questionnaires online, or in cases where internet connectivity is limited, paper-based versions of the questionnaires will be available. The first assessment will take place at baseline, followed by the second assessment after the first refill. The third assessment will occur after the second refill, the fourth after the third refill, and the fifth and final assessment is scheduled following the fourth refill. Additionally, four weeks after the first refill and four weeks after the second one, a health economics telephone interview will take place. Table 2 gives an overview of the specific questionnaires for each assessment. Patients unable to complete self-reported questionnaires independently may receive assistance from caregivers, or the caregiver may complete the assessment on their behalf. Caregiver involvement will be systematically documented, and this information will be considered in data analysis to ensure transparency and maintain data validity.

#### Plans to promote participant retention and complete follow-up.

At each refill, an appointment will be scheduled for the next one. All home-refills will be scheduled to the patient’s wishes. To support participant retention and ensure completion of study procedures, patients will receive a reminder of their upcoming refill appointment a couple of days before. Following each refill, they will be given a tablet with the relevant questionnaires, in the presence of the researcher.

### Data management

#### Data capture system.

An electronic data capture system, REDCap, will be used for data collection. The system is validated and access to all levels will be granted/revoked by the sponsor representative. To minimize missing data, an error message will be displayed if a question is left unanswered.

Collected data will include responses to validated questionnaires assessing patient comfort, QoL, pain intensity, pain interference, anxiety, and self-efficacy. In addition, caregiver burden will be assessed using a validated questionnaire. Finally, demographic information, as well as data on patient satisfaction, safety, and treatment preference, will be collected.

Trial data should be entered within a reasonable time after the refill procedure. Corrections/modifications will be automatically tracked by an audit trail detailing the date and time of the correction and the name of the person performing the correction.

#### Data storage and responsibilities.

During the research period, data management and storage will be overseen by the pre-doctoral investigators. All collected data will be securely stored on a dedicated, system-encrypted SharePoint or Pixiu page at Vrije Universiteit Brussel, with access limited to the investigators and supervisors. A secure external hard drive will be used as an additional backup.

Upon completion of the study, responsibility for the data will transfer to the principal investigator, and all files will be migrated to the Vrije Universiteit Brussel Archive, where they will be retained for 25 years. Before archiving, any direct personal identifiers will be permanently removed.

#### Data protection.

Personal data will be processed in accordance with applicable EU regulations, including the GDPR, the Belgian Law of 30 July 2018 concerning the protection of personal data, and in alignment with Good Clinical Practice (GCP) guidelines.

Pseudonymization will be applied as soon as data collection begins. As an added security measure, the file linking pseudonyms to the original direct identifiers will be encrypted before uploading it to Pixiu/SharePoint.

#### Data security and access control.

Specific measures will be taken to prevent unauthorized access, as personally identifiable information will be collected. Data will be gathered using REDCap, a secure web-based application for data capture. To enhance data protection, questionnaire responses will be accessible only to the study team and secured by password protection.

Personally identifiable data and clinical trial data will be stored separately; the latter will be linked solely through a unique participant ID. Access to informed consent forms, personally identifiable information, and the linkage key between identifiers and participant IDs will be restricted to investigators and supervisors and will be stored separately from the trial data.

#### Metadata and documentation.

Vrije Universiteit Brussel adheres to the FOSB metadata standard, developed by the Flemish Open Science Board, which is compatible with the international DataCite metadata schema.

At the project level, essential metadata (including the project title, investigators, objectives, hypotheses, funding information, study protocol, sampling procedures, data collection instruments, and technical infrastructure (hardware and software)) will be documented and made available through research plans and publications. At the database level, an inventory of the data files will be provided via a comprehensive read-me file. At the data level, a codebook will accompany the dataset, outlining the structure and meaning of quantitative variables, along with the scripts used for data analysis.

#### Confidentiality.

Participant identification codes will be used to pseudonymise data. The file containing the key that links participant numbers to personal data will be securely managed by the research team and will not be accessible to others. As an additional security measure, this key file will be encrypted before being uploaded to SharePoint.

### Statistical methods

#### Statistical methods for primary and secondary outcomes.

Therapy outcomes will be assessed and compared using mixed model analysis. The ordinal nature of the GCQ outcome will be explicitly accounted for by using a proportional odds mixed-effects model, which appropriately handles ordinal outcome data while accounting for within-subject correlation due to repeated measurements. For continuous outcomes, linear mixed-effects models will be used. For all models, the necessity of including random intercepts and random slopes, as well as the assumption of linearity, will be systematically evaluated. In addition, alternative variance-covariance structures will be compared using restricted maximum likelihood tests and Akaike Information Criterion values. If the unstructured model differs not significantly from a model with more assumptions, we will replace it.

To control for potential confounders, baseline measures of stress and anxiety will be incorporated into the model. Differences will be considered statistically and clinically significant at a threshold of α < 0.05. Additionally, based on baseline data, predictive modelling will be conducted to identify which patients will benefit the most from home-based refill procedures. This will involve the application of both supervised and unsupervised machine learning techniques (K-means clustering, decision trees, and Random Forest algorithms). All statistical analyses will be performed in SAS, SPSS and R.

#### Who will be included in each analysis.

All randomized participants will be included in each analysis.

#### Methods in the analysis to handle protocol non-adherence and any statistical methods to handle missing data.

To minimize missing data, questionnaires will be administered electronically via REDCap, requiring completion of all items prior to submission. When data are missing (e.g., due to participant or technical issues), paper-based completion or direct participant contact will be used. All missing data will be documented and entered into the electronic Case Report Form (eCRF).

Analyses will be conducted according to the intention-to-treat principle to assess the effect of the intervention in the full randomized population, thereby preserving the benefits of randomization and supporting the robustness of the results. In addition, per-protocol analyses will be performed to assess the consistency of findings across analysis populations and to evaluate the impact of protocol non-adherence.

Analyses will initially be based on observed data. Linear mixed models will be applied, which allow inclusion of participants with at least one valid measurement, and therefore intermittent missing values are not expected to substantially bias the findings.

To assess the potential impact of missing data and possible informative dropout, a sensitivity analysis will be conducted used multiple imputation by chained equations, incorporating relevant baseline characteristics and outcome variables.

#### Methods for any additional analysis.

Baseline data will provide a cross-sectional overview of general comfort, QoL, pain intensity, pain interference, stress, anxiety, self-efficacy, and caregiver burden in patients receiving IDD. In addition to a descriptive summary of this population, correlation analysis will be conducted to explore the relationships between these outcome measures. If a linear relationship is present between two variables, Pearson’s correlation coefficient will be used. If the assumption of linearity is not met, Spearman’s rank correlation will be applied instead. All correlations will be tested at a significance level of α < 0.05.

For transparency, whether questionnaires were completed by the patient or with caregiver assistance (proxy completion) will be recorded. Although not explicitly detailed in the original protocol, proxy-completed assessments will be explored in sensitivity analyses to evaluate the robustness of outcome reporting.

#### Health economic analysis.

A within-trial economic evaluation will compare the cost-effectiveness of home-based versus clinic-based intrathecal pump refills. Each participant will receive one refill at home and one at the outpatient clinic, with a 2–3 month follow-up between procedures. The analysis will include all participants up to the third refill.

Data on resource use will be gathered via telephone interviews, as outlined in the ‘health expenditure’ outcome section, and valued using official Belgian national tariffs.

Health outcomes will be assessed in two ways: (1) Percentage increase in comfort, as measured by the GCQ. (2) Utility values, derived from EQ-5D-5L questionnaires and converted using Belgian population-based value sets, in line with national health economic guidelines [20]. The outpatient refill procedure will serve as the control group. Any missing data will be addressed before analysis using appropriate methods for handling incomplete health economic data [44].

Cost differences between the two groups will be analysed using generalized linear models. The Modified Park Test will guide the selection of the appropriate model specification [45]. The main result will be the Incremental Cost-Effectiveness Ratio (ICER), calculated as the difference in costs divided by the difference in effects. Effects will be expressed as percentage improvement in functioning and as quality-adjusted life years (QALYs) gained [46].

To address uncertainty in the analysis, a probabilistic sensitivity analysis will be performed [44]. Variability in cost and outcome estimates will be explored through non-parametric bootstrapping. The results will be presented using Cost-Effectiveness Acceptability Curves (CEACs), which illustrate the probability that the intervention is cost-effective across a range of willingness-to-pay (WTP) thresholds. All findings will be reported in accordance with the Consolidated Health Economic Evaluation Reporting Standards (CHEERS) guidelines [47].

In addition to the within-trial economic evaluation, a model-based analysis will be conducted to estimate the long-term costs and health outcomes of the intervention (home-based refills) versus the control (outpatient refills) beyond the trial follow-up period. A Markov model will be developed in accordance with established health economic modelling guidelines. The model will use a one-year cycle length and a lifetime horizon to capture all relevant long-term effects. Lifetime incremental costs and QALY’s will be calculated for both the home-based and outpatient refill procedures. Discount rates of 3% for costs and 1.5% for utilities will be applied, this is in line with the Belgian health economic guidelines [48,49].

The model will include probabilistic sensitivity analysis to account for parameter uncertainty, following the same approach as the within-trials evaluation. Again, results will be visualized using CEACs to illustrate the probability that the home-based intervention is cost-effective at various WTP thresholds. All modelling results will be reported in line with the CHEERS reporting standards [47].

#### Willingness-to-pay.

In addition to the cost-utility analysis, which focuses on QoL and life-years gained, a DCE will be conducted to estimate the willingness-to-pay (WTP) for receiving pump refills at home. This preference-based method will provide insights into the broader societal value of the intervention and support future reimbursement policy decisions [50]. The results will complement the health economic evaluation by providing insight into preferences and perceived value from a societal perspective [51].

A literature review has been conducted to obtain the attributes that represent the patient-centred values relevant to DCE [52–62]. Table 3 represents the attributes with corresponding levels.

**Table 3:** Attributes and corresponding levels used in the DCE in the IMPROVE-study.

Attributes	Levels
Travel time + waiting time	• 0 minutes • 45 minutes • 90 minutes
Person who performs the refill	• Always the same person • Team of 2 persons (alternating) • Always different
Risk of wound infection	• 1 in 100 refills • 1 in 500 refills • 1 in 1.000 refills
Additional required amount that you should have to pay (non-reimbursed)	• € 0 • € 40 • € 70

These attributes will be presented in a choice set consisting of two scenarios (A and B), each representing different combinations of attribute levels. Patients will choose between the two scenarios. The DCE, consisting of thirteen choice sets, will be completed once after the fourth refill. An example of a choice set is shown in Table 4.

**Table 4:** Example of a DCE choice set asking participants “Which scenario do you prefer for your refill procedure?”.

	Scenario A (refill at home)	Scenario B (refill at the hospital)
Travel time + waiting time	There is no extra travel time or waiting time.	Your total travel and waiting time is 90 minutes.
Person who performs the refill	Every refill is performed by a different person.	Every refill is performed by the same person.
Risk of wound infection	The risk of a wound infection for you is 1 in 500 refills.	The risk of a wound infection for you is 1 in 1.000 refills.
Additional required amount that you should have to pay (non-reimbursed)	The cost for you is 40 euros.	The cost for you is 0 euros.

#### Interim analysis.

No interim analysis is planned.

### Monitoring

#### Composition of the coordinating site and trial steering committee.

**The Steering Board.** The Steering Board is the main decision-making and steering body of the project and consists of M.M., L.G., and K.P.. For any decisions to be considered binding, all members (or their appointed substitutes) must be present. At the start of the project, the Steering Board will organize a kick-off meeting to establish common working procedures. Regular meetings will take place every three months, with additional teleconferences scheduled on an ad hoc basis if urgent matters arise.

The main tasks of the Steering Board are: 1) installing an agreement on common working procedures and management policies; 2) monitoring overall project progress and the timely delivery of all deliverables; 3) assessing if milestones have been reached; 4) deciding on major changes to the work programme if necessary; 5) conflict handling, including referral to the Conflict Council when necessary; and 6) overseeing budget-related decisions. One of the most important tasks of the Steering Board is to ensure that patients follow the refill schedule correctly and complete all questionnaires in a timely manner. More broadly, the Steering Board is accountable for maintaining the quality of the workflow and the overall implementation of the project, while taking into consideration the available resources.

**The Conflict Council.** The Conflict Council is part of the management structure and will only be activated in case of a conflict. The Conflict Council will consist of 5 members from our University in total and will convene face-to-face to discuss the conflict matter(s) at hand and will decide on conflict resolution by simple majority.

**The Advisory Committee.** The Advisory Committee (or valorisation board) consists of the project coordinator, a representative from each project partner and the stakeholders who agreed to take part in the project.

#### Composition, role, and reporting structure of the data monitoring committee.

The study coordinator at VUB will regularly monitor data that are entered in REDCap and in the Excel file for the functional capacity evaluation. The study coordinator is independent from the funder of this study and has no competing interests.

#### Trial monitoring.

The study staff will submit an annual progress report to the Ethical Committee (EC), including enrolment dates, subject numbers, trial completions, SAEs/reactions, issues, and amendments. No on-site audits are planned, but an independent quality assurance representative may review the study to ensure regulatory compliance. Auditors may inspect the sites anytime during or after the trial and access all study data, source documents, and patient files.

#### Protocol amendments.

Before implementation, all protocol amendments will require approval from the EC. Patients will be informed of any relevant changes to the protocol.

#### Ancillary and post-trial care.

There are no planned ancillary studies or no post-trial care is provided. No harm is anticipated from trial participation; therefore, no compensation for potential harm has been foreseen.

## Discussion

The safety and feasibility of performing refills in the home environment has already been demonstrated in previous studies, confirming that such procedures can be conducted effectively and without increased risk of complications [4,12,13]. The pilot findings indicated high patient satisfaction and overall acceptance of the procedure, aligning with the broader development of HAH programs [12]. These initiatives have expanded substantially since the COVID-19 pandemic, offering a viable alternative to traditional inpatient care [63]. Home-based refills through HAH may help reduce the burden, stress, and pain caused by hospital visits for both patients and their informal caregivers [4].

Prior studies have shown that HAH programs can improve patient QoL, delay readmissions, and reduce healthcare costs [64–66]. However, the long-term clinical and economic effects of such models remain insufficiently understood [67]. The IMPROVE study will investigate whether performing intrathecal pump refills at home leads to differences in patient comfort compared to refills in a hospital setting. If home-based refills demonstrate greater patient comfort than standard hospital care, HAH should be considered the new standard conservative approach for these patients. In addition to comfort, the trial will also assess secondary outcomes including safety, QoL, clinical effectiveness, and cost-effectiveness to provide a comprehensive understanding of the potential impact of this care model in routine practice.

Hence, before widespread implementation of home-based intrathecal pump refills can be recommended, several factors still require careful evaluation. Variability in home environments, patient living conditions, and workflow processes may challenge the wider adoption of HAH interventions. To address these factors, the study includes an embedded implementation evaluation focusing on the role of telemedicine and interdisciplinary collaboration.

Socioeconomic conditions, geographic location, caregiver availability, and cultural attitudes toward receiving medical care at home may influence the feasibility and acceptance of home-based intrathecal pump refills. Differences between urban and rural environments, along with variations in health literacy and social support, can influence both implementation and the patient experience. As the study includes adult patients across a broad age range, variability in experiences related to autonomy, caregiver dependence, and home-based care preferences may already be partially captured within the study population.

Because this study is conducted within a specific healthcare system, its findings may have limited generalizability to other healthcare contexts or patient populations. The study is not specifically designed or powered to evaluate socioeconomic or cultural determinants. However, relevant contextual characteristics of participants, including socioeconomic and demographic variables, will be collected to inform interpretation of study findings and to guide future implementation research.

From an implementation perspective, several practical factors may influence the adoption of home-based intrathecal pump refills. These include staff training requirements, the availability and cost of devices, and the logistical organization of home visits, such as geographical coverage, and scheduling. In the present study, procedures were performed by experienced healthcare professionals, and additional training requirements were limited. However, these factors may vary across healthcare systems and should be considered in future implementation and cost-effectiveness studies.

Future research could be based on the current study by conducting larger multicenter trials with longer follow-up to evaluate sustained clinical, patient-reported, and economic outcomes. Additional studies may assess the influence of contextual factors such as socioeconomic status, caregiver availability, health literacy, and cultural attitudes on feasibility and acceptance across diverse healthcare settings. Moreover, practical aspects of home-based refills, including workflow optimization and ergonomics for healthcare professionals, warrant further investigation, as patients’ choice of refill location within their home can affect the comfort and efficiency of the procedure performed by the physician. Taken together, this line of research will establish a solid evidence base to support the safe, patient‑centered, and sustainable integration of home‑based intrathecal pump refills into routine clinical practice.

In summary, building on existing evidence and addressing implementation and contextual considerations, this trial represents an important next step toward making at-home intrathecal pump refills part of routine care. The study looks at clinical outcomes, costs, and how the procedure can be carried out in practice, to provide evidence for wider use of HAH for pain and spasticity management.

### Trial status

Recruitment has started in October 2025 and will be ongoing until 82 patients are included in the study. The recruitment phase is anticipated to last 28 months, targeting an average inclusion rate of three patients per month. The current protocol is version 3 of October 2025.

## Supporting information

S1 File SPIRIT 2025 checklist. Checklist of items to address in a randomized trial protocol.
(PDF)

S2 File IMPROVE protocol. Original protocol approved by the ethics committee.
(PDF)

S1 Fig Graphical abstract of the IMPROVE-study. Summary of the study design, objectives and expected outcomes.
(PDF)

S2 Fig Intrathecal pump refill protocol. Step-by-step protocol of all procedures involved in intrathecal pump refills, including patient positioning, pump readout, preparation of prescribed medication, skin disinfection, aseptic access to the pump reservoir, intrathecal pump refill, and needle removal.
(PDF)

S3 Fig Differences between hospital- and home-based pump refills. Comparison of the procedures according to the refill setting, type of supervision, verification process, and follow-up.
(PDF)

S4 Fig Emergency management flowchart. Flowchart for the management of (severe) complication during home intrathecal pump refills.
(PDF)

S5 Fig Implementation checklist for home-based intrathecal pump refills. Checklist covering pre-visit preparation, required materials, home environment setup, the refill procedure, post-procedure care, and follow-up.
(PDF)

S1 Table Comparison of expected clinical outcomes. The expected outcomes between home and hospital refill settings are compared.
(PDF)

## Abbreviations

IDD: intrathecal drug delivery
BBB: blood-brain-barrier
HAH: hospital at home
HRQoL: health-related quality of life
((S)AEs): (Serious) adverse events
REDCap: research electronic data capture
DCE: discrete choice experiment
GCQ: general comfort questionnaire
MQOL: McGill quality of life questionnaire
EC: ethics committee
EQ-5D: EuroQol with five dimensions
VAS: visual analogue scale
PROMIS: patient-reported outcomes measurement information system
STAI: state trait anxiety inventory
GSE: general self-efficacy
ZBI: Zarit Burden interview
DCE: discrete choice experiment
NIH: National Institutes of Health
HPA: hypothalamo-pituitary-gland
ARs: adverse reactions
CA: competent authorities
SUSARs: suspected unexpected serious adverse events
EU: European open
GDPR: general data protection regulation
GCP: good clinical practice
ICER: incremental cost-effectiveness ratio
QALYs: quality-adjusted life years
CEACs: cost-effectiveness acceptability curves
CHEERS: consolidated health economic evaluation reporting standards
WTP: willingness-to-pay
EC: ethical committee
eCRF: electronic Case Report Form
