# Supplementary material for: Intrathecal pump refills at home or at the hospital: Protocol for a randomized controlled crossover trial—The IMPROVE study
Source: PLoS One. 2026 Jul 27;21(7):e0354092. doi: 10.1371/journal.pone.0354092 (PMC13405089; doi:10.1371/journal.pone.0354092)
Supplement: S2 File — Original protocol approved by the ethics committee. (PDF) [file pone.0354092.s002.pdf]

Study Protocol

Study Title: *Intrathecal pump refills at home or at the hospital: a randomized controlled crossover trial*

Study Acronym: *IMPROVE*

Protocol Version and Date: IMPROVE Version *2.0 - September 2, 2025*

Registry Number (*if applicable*): *ClinicalTrials.gov number not yet known*

Sponsor: *Vrije Universiteit Brussel*

Coordinating/Principal Investigator: *Prof. Dr. Maarten Moens*

Study Start Date: *11/SEPT/2025*

Expected Study End Date*: *01/OCT/2028*

The information contained in this document is the property of the Sponsor/Coordinating Investigator and may not be reproduced, published or disclosed to others without written authorization of the Sponsor/Coordinating Investigator.

PROTOCOL SIGNATURE PAGE

Name	Signature	Date
**Principal Investigator:**<br>*Prof. Dr. Maarten Moens, STIMULUS research group VUB, department of Radiology/Neurosurgery UZB*		

Table of Contents

PROTOCOL SIGNATURE PAGE 1

Principal Investigator: 1

1 Sponsor/Coordinating Investigator Information 4

2 List of Abbreviations 5

3 Protocol Version History 5

4 Trial Registration/Protocol Summary 5

5 Background and Rationale 7

5.1 Overview of Relevant Literature 7

5.1.1 Burden of chronic pain 7

5.1.2 Pain management: burden of systemic opioids 7

5.1.3 Intrathecal drug delivery (IDD) as a solution 7

5.1.4 Performing pump refills at home; a clinical relevant PoC study 8

5.1.5 Need for head-to-head comparison and economic evaluation 8

5.2 Study Rationale and Purpose 9

6 Study Objectives and Endpoints 9

6.1 Primary Objective 9

6.2 Secondary Objectives 9

6.3 Endpoints 10

6.3.1 Primary outcome 10

6.3.2 Secondary outcomes 10

7 Study Design 13

7.1 Study Design and patient enrollment 13

7.2 Randomization 13

7.3 Blinding 13

7.4 Date Range for collected study data 13

8 Study Population 14

8.1 Population of interest 14

8.2 Inclusion Criteria 14

8.3 Exclusion Criteria 14

9 Study Assessments and Procedures 14

9.1 Schedule of Activities 14

9.2 Detailed Study Assessments/Interventions 15

10 Data Collection and Management ... 16

10.1 Monitoring ... 16
10.1.1 Adverse events and adverse reactions ... 16
10.1.2 Serious adverse event or reaction ... 17
10.1.3 Suspected unexpected serious adverse reaction (SUSAR) ... 18
10.1.4 Procedures for handling special situations ... 18

10.2 Data Collection ... 19

10.3 Database Management and Quality Control ... 19

10.4 Statistical Considerations and Data Analysis ... 20
10.4.1 Baseline analysis ... 20
10.4.2 Main analysis ... 20
10.4.3 Health economic analysis ... 20

11 Ethical Considerations ... 22

11.1 Ethical Conduct of the Study ... 22
11.1.1 Declaration of Helsinki ... 22
11.1.2 Ethics Committee (EC) ... 22

11.2 Recruitment and Informed Consent (IC) ... 22

11.3 Sample size calculation ... 23

11.4 Study Data Protection ... 23

11.5 Subject Identification ... 23

12 Insurance ... 24

13 Reporting and Dissemination ... 24

14 Finance and Conflict of Interest Statement ... 24

15 Tables and Figures ... 25

16 References ... 27

Sponsor/Coordinating Investigator Information

Sponsor	**Vrije Universiteit Brussel**<br>*Laarbeeklaan 103, B-1090 Jette, Belgium*
Coordinating/Principal Investigator	**Prof. Dr. Maarten Moens**<br>*UZ Brussel – Department of Radiology/Neurosurgery*<br>*Vrije Universiteit Brussel – Stimulus Research Group*
Co-investigators	**Prof. Dr. Lisa Goudman**<br>*UZ Brussel - Department of Neurosurgery*<br>*Vrije Universiteit Brussel – Stimulus Research Group*<br><br>**Prof. Dr. Koen Putman**<br>*Vrije Universiteit Brussel - Interuniversity*<br>*Centre for Health Economics Research (I-CHER)*<br><br>**Dra. Ulrike Van Hoey**<br>*Vrije Universiteit Brussel – Stimulus research group*<br><br>**Dra. Britt Winnepenninckx**<br>*Vrije Universiteit Brussel – Stimulus research group*
Statistician	**Prof. Dr. Lisa Goudman**<br>*UZ Brussel - Department of Neurosurgery*<br>*Vrije Universiteit Brussel – Stimulus Research Group*
Pharmacy	*Hospital pharmacies of UZ Brussel*

List of Abbreviations

AE	Adverse Event
AR	Adverse Reaction
ATMP	Advanced Therapy Medicinal Product
CA	Competent Authority
CRF	Case Report Form
CT	Clinical Trial
DCE	Discrete Choice Experiment
DSMB	Data Safety Monitoring Board
EC	Ethics Committee
e-CRF	Electronic Case Report Form
EU	European Union
GCP	Good Clinical Practice
GDPR	General Data Protection Regulation
HAH	Hospital at Home
IC	Informed Consent
ICF	Informed Consent Form
IDD	Intrathecal Drug Delivery
PI	Principal Investigator
SAE	Serious Adverse Event
SAR	Serious Adverse Reaction
SUSAR	Suspected Unexpected Serious Adverse Reaction

Protocol Version History

Version N°	Version Date	Summary of changes
1.0	25/06/2025	NA
2.0	02/09/2025	- On p.8-9, section 5.1.5.: explanation regarding who the "trainee" is and the "researchers at home and in the hospital."<br>- On p.15, section 9.2.: suggestion for presence of a caregiver during 12 hours after the refill period.<br>- On p.10, under section 6.3.: the concrete procedure was explained to carry out the cost analysis.
3.0		- On p.4: Name of Jocelyn Castille was deleted<br>- On p.11, section 6.3, Table 1, and p.23 section 10.4.3: DCE will only be asked after the fourth refill, not at baseline.<br>- On p. 22, Table 2, and Table 3: The DCE includes four attributes instead of six, each with three levels.<br>- On p.12 section 6.3.2.: Change of kit used to analyse cortisol level to 'Cobas 8000: Elecsys Cortisol II, electrochemiluminescence immunoassay'

Trial Registration/Protocol Summary

**Information**	
Objectives:	The **primary objective** of this study is to evaluate if intrathecal pump refills at home are more comfortable compared to intrathecal pump refills at the hospital.<br>The **second objective** is to evaluate if there is a difference in health-related quality of life, pain intensity, pain interference, stress, anxiety, general self-

	efficacy, burden among caregivers, patient preference for the location of the refill and in number of (serious) adverse events (e.g., infection, overdose).<br>The **third objective** is to conduct an economic evaluation of intrathecal pump refills at home versus at the hospital, based on healthcare expenditure and (in)direct costs.
Study population:	Patients with chronic non-cancer pain and patients with spasticity, who were previously implanted with an implantable pump for intrathecal drug delivery.
In- and exclusion criteria:	**Inclusion:**<br>- Adult patients ≥18 years<br>- Actively receiving Intrathecal drug delivery (IDD)<br>- Stable medication dosage for at least 3 months<br>- Dutch, French or English speaking<br><br>**Exclusion:**<br>- Life expectancy < 6 months<br>- Patients planned for but not yet received IDD implant<br>- No residence in Belgium
Data to be collected:	- Patient comfort – General Comfort Questionnaire (GCQ)<br>- Quality of Life (QoL) – McGill Quality of Life Questionnaire (MQOL) and EuroQol (EQ-5D-5L)<br>- Pain intensity – Visual Analogue Scale (VAS)<br>- Pain interference – Patient-Reported Outcomes Measurement Information System<br>- Stress – saliva samples with synthetic salivettes (Sarstedt AG & Co., Nümbrecht, Germany)<br>- Patient anxiety – State Trait Anxiety Inventory (STAI)<br>- Self-efficacy – General Self-Efficacy (GSE) Scale<br>- Caregiver burden – Zarit Burden Interview (ZBI)<br>- Patient satisfaction – Seven-point Likert-scale<br>- Tele-monitoring quality<br>- Time of the refill<br>- Safety<br>- Health expenditure<br>- Patient preference
Endpoints:	The **primary endpoint** is to observe whether there is a difference in the scoring of the General Comfort Questionnaire between patients with chronic non-cancer pain or spasticity after receiving a pump refill at home versus in the hospital. Each patient will receive two pump refills at home and two in the hospital.<br>The **secondary outcome** is whether the two arms differ on quality of life (QoL), pain intensity, pain interference, stress, anxiety, self-efficacy, burden of caregivers for patients who receive IDD, patient satisfaction, consultation time, safety, health expenditure, and patient preference. A mixed model analysis will be used with timepoints at baseline and after each refill.
Target sample size:	N ≥82
Statistical considerations:	Using baseline data, cross-sectional results will be provided on general comfort, quality of life (QoL), pain intensity, pain interference, stress, anxiety, self-efficacy, and burden of caregivers for patients who receive IDD. In addition to describing this population, supervised and non-supervised

	machine learning techniques will be applied such as hierarchical clustering, mediation analysis and regression models.<br>To evaluate and compare therapy effects, mixed model analysis will be used.

Background and Rationale

Overview of Relevant Literature

Burden of chronic pain

Chronic pain is commonly defined as pain that lasts beyond 3 months and extends past normal tissue healing time. Both chronic primary pain syndromes—recognized as distinct health conditions in which pain cannot be attributed to another chronic pain disorder (1) – and chronic secondary pain syndromes – where pain is considered a symptom of an underlying disease (2) – are included under the umbrella of chronic pain in the 11th Revision of the International Classification of Diseases (ICD-11). Estimates of the prevalence of chronic pain range between 20% and 50%, indicating that chronic pain affects between 1 in 5 to 1 in 2 people (3–5). The variation across studies is mainly due to different definitions of chronic pain and the exact populations studied (4).
Chronic pain is more prevalent than many other chronic conditions, including diabetes mellitus (14.1%; 95% CI: 13.4–14.7) and rheumatoid arthritis (0.5%; 95% CI: 0.3–0.9) (6,7). Chronic pain presents a significant health burden associated with significant reductions in health-related quality of life (8).

Pain management: burden of systemic opioids

Chronic pain management typically begins with strategies that are least invasive and carry the lowest risk of complications. When initial, lower-risk treatments fail to provide adequate relief, the management plan progressively incorporates more advanced interventions that carry greater risks (9). Although the use of opioids is controversial and currently recommended for only a short period of time, opioids are still a valuable world-wide option in the armamentarium of clinicians treating patients with chronic pain (10). Despite recommendations and guidelines to avoid doses above 90 MME (morphine milligram equivalents) (11,12), up to 39.5% of chronic non-cancer pain patients receive doses ≥ 90 MME (13). Belgium still belongs to the top three European countries in defined daily dose analgesic opioids use (14). Long-term opioid use is associated with constipation, hyperalgesia, respiratory depression, sedation, increased risk of gastrointestinal adverse events, addiction and increased risk of vomiting, while patients can become tolerant (15). Consequently, a complex clinical challenge has surfaced as tapering of systemic opioids is likely to increase patient's pain intensity, whereas further increasing systemic opioid doses will aggravate the addition, tolerance, adverse events and pain sensitivity (16).

Intrathecal drug delivery (IDD) as a solution

Intrathecal analgesia represents a therapeutic option for patients with refractory pain or those who experience intolerable side effects from conventional treatments. Intrathecal drug delivery (IDD) is often considered a last-resort intervention for individuals who have endured prolonged pain – typically over several years – and who fail to achieve sufficient analgesia despite high-dose opioid therapy (17). Therefore, intrathecal analgesia may be adopted for non-oncologic pain in patients with compression fractures, spondylolisthesis, spondylosis, persistent spinal pain syndrome type II and spinal stenosis (18). Other conditions include spinal cord injury-induced spasticity and neuropathies (19).
During a surgical procedure, a small pump is placed under the abdominal skin and delivers pain medication through a catheter to the area around the spinal cord. Intrathecal drug delivery allows targeted and continuous drug infusion directly into the intrathecal space, thus bypassing the blood-brain barrier (BBB) (18). The BBB represents the major obstacle to the administration of drugs into the

central nervous system and delays the passage of up to 98% of systematically administered drugs into the cerebrospinal fluid (18,20). Thus, with drug administration in the vicinity of the action site, a much lower dose of medication is required than with systemic administration routes. Consequently, opioid serum levels are minimal or undetectable, and side effects may be reduced (18,21). It is already demonstrated that IDD is an effective and safe management option that provides significant and sustained pain relief for patients with spasticity, non-cancer pain and cancer pain, and can result in a reduction of systemic opioid consumption by on average >50% (17,21,22).

Performing pump refills at home; a clinical relevant PoC study

Hospital-at-home (HAH) is a home-based alternative for acute care that has expanded significantly under COVID-19 regulatory flexibilities (23). Several trials concerning HAH modalities, including supportive oncology care at home, telemedicine, symptom management programs and remote monitoring, have been conducted in the past two years (24–27). In patients with heart failure, a meta-analysis indicated that HAH significantly increased time to first readmission and improved health-related quality of life compared to routine hospitalization (SMD -0.31 (95% CI from -0.45 to -0.18) at 6 months and -0.17 (95% CI from -0.31 to -0.02) at 12 months) (28). A Cochrane review concerning the effectiveness of home-based end-of-life care supported improved patient satisfaction in the short term (29). Additionally, costs of hospitalization were reduced as well with home-based therapy (30,31).

In case of IDD, after pump implantation, the most performed postoperative maintenance procedure is the drug refill, consisting of an aseptic access to the pump reservoir, emptying residual volume, and filling the reservoir with new medication (32). Successful therapy thus requires continuous follow-up by specialists, as pump refills need to be performed at regular time intervals, while it necessitates patients to get refilled in an outpatient hospital setting (33). For both specialists and patients, the process is burdensome and entails pitfalls as delayed pump refill can cause serious withdrawal syndrome and, in case of baclofen, life-threatening events may occur such as rhabdomyolysis, seizure, coma and even death (34). Consequently, intrathecal pump refills and intrathecal pump malfunctioning are allocated as urgent patient procedures (35).

In 2021, we demonstrated that intrathecal pump refill procedures can be performed in a HAH setting, whereby effectiveness, safety and feasibility have been demonstrated (36). In our PoC (Proof of Concept) study, 20 patients received a pump refill at home, whereby all patients indicated they were satisfied with this approach (36). The same conclusions were drawn from a retrospective analysis of pump aftercare in the Netherlands, including refill on location (37,38). Via HAH refills, the patient's and informal carer's burden, stress and increased pain from travelling to the hospital can be avoided. Our PoC already identified barriers, while in 2023 and 2024, we further assessed and confirmed the burden of outpatient hospital refills on patients and their caregivers. Of a convenience sample of 41 patients who received IDD at our hospital, 21 were accompanied by an (in)formal caregiver of whom 42.8% had to ask for caregiver hours at work and 1 patient had to take time off from work for the refill procedure at the hospital. Of the 41 patients, 17% came to the hospital by ambulance or adapted patient transport (preliminary data, not published).

Need for head-to-head comparison and economic evaluation

Systematic reviews remain unclear about the economic benefit of HAH programs (28,39). To comprehensively assess the economic impact of HAH, several factors should be considered, including the involvement of specialists, the feasibility of multi-session home treatments, the hospital's location and financial status, as well as the geographical distribution of patients (40). However, advances in technology have paved the way for the inclusion of telemedicine in surgery and acute management (41,42). The initial development focused on telepresence, enabling the remote operating site to be experienced in a natural and immersive way, thereby creating a sense of presence. Ongoing research continues to demonstrate the effectiveness of telemonitoring and remote telesurgery, where an experienced surgeon can conduct procedures from a distance, through for example robots (43). In our PoC study in performing pump refills at home, both the physician and specialist nurse conducted refill procedures at home. This application fits well with the goals of telemonitoring and telemedicine, making it useful for remote pump refills.

A proper economic evaluation to determine the cost effectiveness as well as a full evaluation of patient-reported outcomes concerning health-related QoL and emotional and psychological well-being have not yet been conducted. This is, however, crucial for further implementation of remote refill procedures in clinical practice and seems to be the final hurdle that must be taken to provide the HAH option to patients. A utility score of 0.4523 for a successful refill at the hospital and of 0.4658 for an intrathecal refill procedure at the patient's home is expected (assumption 3% higher) (44–49). We conducted a back-of-the-envelope calculation that resulted in an incremental cost effectiveness ratio (ICER) of €32.255/quality-adjusted life year (QALY) and €12.148/QALY on a 10-year time horizon for 4 versus 6 IDD refills per year respectively (depending on the exact dose of medication 4 or 6 refills per year are needed), demonstrating that refills at home have the potential to be cost-effective.

Study Rationale and Purpose

With IMPROVE, we propose to perform intrathecal pump refills at the patient's home instead of at the hospital, in a population of patients with chronic pain or severely disabling spasticity. In the past, a promising PoC regarding the safety and feasibility of out-hospital intrathecal pump refills in chronic pain patients was already gathered (36). Despite the positive responses in terms of patient satisfaction, a head-to-head comparison of refill procedures at the hospital or at home and their cost effectiveness has yet to be conducted. With IMPROVE, we want to build on our PoC and nationally deliver an intervention that not only increases patient comfort but also proves to be cost effective for society.

Therefore, we propose to start with intrathecal pump refills at the patient's home instead of at the hospital for patients implanted with an intrathecal pump. This allows us to tackle the high burden of patients that experience increased pain due to travelling, are dependent on third parties for transport and are confronted with additional stress due to the hospital visit by proceeding towards a more logical long-term treatment plan for a debilitating condition. Due to current reimbursement rules in Belgium, only patients with chronic non-cancer pain or spasticity who receive IDD are eligible to participate.

Study Objectives and Endpoints

Primary Objective

The primary objective of this study is to evaluate if intrathecal pump refills at home are better for patient comfort compared to intrathecal pump refills at the hospital. The primary outcome measure, i.e. patient comfort, is evaluated with the General Comfort Questionnaire (GCQ).

Secondary Objectives

The secondary objective is to evaluate if there is a difference in health-related quality of life, pain intensity, pain interference, stress, anxiety, general self-efficacy, burden among caregivers, patient preference for the location of the refill and in number of (serious) adverse events (e.g., infection, overdose). The third objective is to conduct an economic evaluation of intrathecal pump refills at home versus at the hospital, based on healthcare expenditure and (in)direct costs.

Endpoints

During the baseline assessment, patients are asked to provide information on their demographic and socio-economic background—including sex, age, marital status, ethnicity, years of education, highest

obtained degree, employment status, income, whether or not they receive home care or have an informal caregiver, and household composition — as well as clinical baseline data, such as the year of pump implantation, medication, indication for having an IDD pump. The general data protection regulation principle of data minimization and proportionality will always be considered.

Further, patients will receive questionnaires related to our primary and secondary outcome measures, as described below. Following this initial assessment, patients will be randomized. The randomization list will be created in R, whereby each patient will receive two refills in the outpatient clinic and two refills at home in a randomized order. After each refill, all baseline outcome measures will be reassessed. In addition, secondary outcomes such as patient satisfaction and preference, telemonitoring quality (applicable only for home refills), and safety will be evaluated during follow-up assessments. Health expenditure data will be collected through phone calls. The researchers will call each patient four weeks after the first refill and four weeks after the second refill. Questions will be asked concerning health-economic aspects. These questions will be based on the following variables: consultations, adverse events, hospitalizations, medication, other health-related costs. For more details concerning these questions, we refer to the health economic telephone interview. To prevent test order effects, the test order of the self-reported measures will be randomized for each individual patient at each assessment. After the fourth refill patients will be asked to complete a Discrete Choice Experiment (DCE). Table 1 (section 15) provides an overview of the outcomes, the timing of assessments, and the designated respondents for each questionnaire.

The self-reported measures will be completed online via REDCap (Research Electronic Data Capture) to enhance study feasibility and ensure data security. For participants who are unable to complete the questionnaires online, or in cases where internet connectivity is limited, paper-based versions of the questionnaires will be available.

Primary outcome

The primary outcome of this study is patient comfort, selected specifically to determine whether HAH care provides benefits for patients with chronic non-cancer pain and spasticity. This concept is grounded in Kolcaba's mid-range theory of comfort, which defines comfort as "The immediate experience of being strengthened by having needs for relief, ease, and transcendence met in four contexts: physical, psychospiritual, sociocultural, and environmental, and it is so much more than the absence of pain" (50,51). Patient comfort is evaluated with the General Comfort Questionnaire (GCQ), a self-reported instrument consisting of 48 items that reflect the physical, spiritual, environmental and social dimension (50). Responses are rated on a four-point Likert scale ranging from "strongly disagree" to "strongly agree". Some items are reverse scored during data processing, with higher overall scores indicating greater levels of comfort (52).

Secondary outcomes

<u>Quality of life (QoL)</u>

The McGill Quality of Life Questionnaire (MQOL) consists of 17 questions. 16 of these are rated on an 11-point Likert scale (range from 0 to 10) and assess various domains including physical symptoms, physical wellbeing, psychological symptoms, perception towards life and sense of achievement (53,54). The final part is an open question. Higher scores reflect a better QoL (53). Originally this questionnaire was developed for palliative patients (54), the MQOL has since been validated for patients with chronic conditions (53). It captures a multidimensional view of QoL by evaluating physical wellbeing, psychological wellbeing, existential wellbeing, social support, and physical symptoms, along with an overall QoL rating (53).

Additionally, the EuroQol with five dimensions and five levels (EQ-5D-5L) will be assessed. Based on the Belgian EQ-5D-5L value set developed by Bouckaert et al. (2022), index scores range from −0.42 to 1 (with 0 corresponding to death and 1 to full health), as derived from population preferences (55).

Pain intensity

The Visual Analogue Scale (VAS - 100 mm) in electronic format will be used for the assessment of current pain intensity. The VAS pain score is reliable, valid, and sensitive to change (56,57). VAS measurements are found to have good test-retest reliability (58).

Pain interference

Pain interference refers to the degree to which pain hinders or restricts an individual's physical, emotional, and social functioning (59). The Patient-Reported Outcomes Measurement Information System (PROMIS ®) was developed to provide a standardized, valid, and reliable measure of clinical outcomes across diverse patient populations (60). The NIH-funded PROMIS Pain Interference measure assesses the impact of pain on key aspects of an individual's life. It evaluates the extent to which pain disrupts engagement in social, cognitive, emotional, physical, and recreational activities. The PROMIS Pain Interference item bank has demonstrated strong psychometric properties, including reliability and construct validity, supporting its use as an outcome measure in chronic pain settings (59).

Stress

Exposure to acute psychological stress triggers two primary physiological systems: the sympatho-adrenal medullary (SAM) system and the hypothalamo-pituitary-adrenal (HPA) axis (61). Activation of the SAM system leads to the rapid release of catecholamines—adrenaline and noradrenaline—which stimulate increases in heart rate and blood pressure (62). This response is also marked by elevated levels of salivary alpha-amylase, a biomarker of sympathetic nervous system activity (61,63). The HPA axis contributes to the longer-term stress response through the release of cortisol from the adrenal glands (61,62,64). Saliva samples will be collected to measure cortisol levels. This provides a non-invasive and valuable method for assessing HPA-axis activity and stress levels (65). These samples will be obtained with synthetic salivettes (Sarstedt AG & Co., Nümbrecht, Germany) 5 min before the start of the refill procedure, immediately after the refill procedure and 10 minutes afterwards. Saliva samples will be stored at -20°C until analysis. The saliva samples will be analysed using the Cobas 8000: Elecsys® Cortisol II, electrochemiluminescence immunoassay.

Patient anxiety

Patient anxiety will be provided through the State Trait Anxiety Inventory (STAI). The STAI, developed by Spielberger, Gorsuch, and Lushene, is designed to provide an objective assessment of anxiety levels in psychologically healthy adults (66). This questionnaire consists of 40 Likert-type items on a four-point scale. STAI is widely used in chronic pain research to assess anxiety, as it captures both acute anxiety symptoms and anxiety as a stable trait of the patient's personality (66,67). For both types of anxiety, the total score ranges from 20 to 80, with higher scores indicating higher levels of anxiety. The STAI is a highly reliable measure that can discriminate between high- and low-stress situations (66).

Self-efficacy

The General Self-Efficacy (GSE) Scale is a 10-item self-report questionnaire used to assess perceived self-efficacy. This construct reflects an individual's belief in their ability to cope with challenging situations and to successfully achieve goals (68,69). In the context of chronic pain, it encompasses not only the expectation of being able to perform specific tasks or behaviors, but also the confidence to do so despite the presence of pain (69–71). Higher self-efficacy scores have shown to be associated with lower pain intensity levels, better physical health-related QoL, better mental health related QoL, lower catastrophizing, and use of coping strategies (70,71). The GSE Scale has been used in chronic pain conditions (72). Its validity was assessed in participants dealing with stressful health-related situations (73).

Caregiver burden

To evaluate caregiver burden, the Zarit Burden Interview (ZBI) questionnaire will be completed (74). This 12-item self-report instrument assesses caregiver burden and is a shortened version of the original 22-item Zarit Burden Scale (75–77). Each item is rated on a 5-point scale, with higher scores indicating greater burden; a score of 4 on any item reflects the highest level of perceived strain. The total score ranges from 0 to 48, with higher totals reflecting more significant caregiver burden. The questionnaire focuses on aspects such as time demands, physical health, mental strain, and psychosocial stressors (75).

Patient satisfaction

Patient satisfaction is evaluated with a seven-point Likert scale asking the patient to rate the overall level of satisfaction with the refill at home.

Tele-monitoring quality

The quality of tele-monitoring will only be evaluated after a home-filling by the researcher at home and the researcher in the hospital. This will be examined using three different Likert scales to score 1) quality of audio, 2) quality of video, and 3) overall quality of the teleconsultation (78).

Time of the refill

The time required for the procedure will be recorded from two perspectives. First, the time for the refiller will be measured: for home-based refills, this includes the duration from entering the patient's house until departure. For hospital-based refills, it covers the time spent in the patient's room in the outpatient clinic. Second, the time required for medical supervision will be recorded: for home-based refills, this includes the full duration of the online consultation. For hospital-based refills, it includes the time the physician spends in the outpatient clinic with the patient.

Safety

Patients will evaluate the overall safety of the procedure using a seven-point Likert scale. In addition, both the researchers (at home and at the hospital) will assess safety on three levels: (1) overall perceived safety of the procedure using a seven-point Likert scale; (2) environmental safety, assessed through an open-ended question addressing any situations perceived as unsafe; and (3) the ability to perform the procedure in a clean and sterile manner, also evaluated via an open-ended question. Between two and twelve hours after the refill at home, the researcher at the hospital will contact all patients by phone to assess the occurrence of any adverse events.

Health expenditure

Expenditures related to in-hospital care will be extracted from hospital claims data. All other healthcare-related costs will be gathered through telephone interviews with patients, conducted four weeks after the first and second medication refills. During these calls, researchers will ask patients whether they have had any medical consultations, hospital admissions, adverse events, changes in medication, or incurred any additional healthcare costs.

Patient preference

After the fourth refill procedure, patients will be asked to indicate their preference (refill at home, refill in the hospital, no preference or other) for the location of their refills in the future. Additionally, patients will be presented with a survey to evaluate their preferences across several scenarios to conduct a willingness-to-pay analysis.

Study Design

Study Design and patient enrollment

The IMPROVE study is a randomized, controlled, cross-over trial to evaluate if intrathecal pump refills at home (Intervention) are better for patient comfort (Outcome) compared to intrathecal pump refills at the hospital (Control) in patients who receive IDD (Population). We will include at least 82 patients, who will each receive two refills in the outpatient clinic and two refills at home.

The physician will briefly explain the IMPROVE study to eligible patients. If the patient is interested, the researcher will explain the study concept and practical aspects of their potential participation. Additionally, the patients will be screened based on in- and exclusion criteria.

The researcher will explain the study to the patient, what's expected for them and what's in it for them. If the patient is positive about participation, IC and contact information will be provided. IC will be obtained on paper. After receiving a signed IC, the IC will be scanned and stored electronically after which the original paper version will be destroyed.

Randomization

The randomization list will be created in R, each patient will receive two refills in the outpatient clinic and two refills at home in a randomized order. The refill procedures are grouped in pairs (one refill at home and one refill in the outpatient clinic) to counter the effect of time-related confounding effects on the dependent variable. This leads to four possible randomization tracks (Fig. 1, section 15).
A list with patient numbers and the group allocation order that results from this randomization procedure will be stored in a sealed envelope. Randomization will take place in the week after the baseline visit.

Blinding

The statistician will be blinded to group allocation. Patients and the researchers performing the refills cannot be blinded to the refill location. To minimize bias, patients will complete the study outcome measures electronically, ensuring that the outcome assessor cannot influence their responses.

Date Range for collected study data

Data collection will take place from 1/AUG/2025 till 1/OCT/2028.

Study Population

Population of interest

Adult patients receiving IDD, regardless of the indication (spasticity, non-cancer pain) and intrathecal drugs used (morphine, baclofen, other).

Inclusion Criteria

- Adult patients, 18 years or older
- Actively receiving IDD
- Stable medication dosage for at least 3 months
- Dutch, French or English speaking

Exclusion Criteria

- Life expectancy < 6 months
- Patients planned for but not yet received IDD implant
- No residence in Belgium

Study Assessments and Procedures

Schedule of Activities

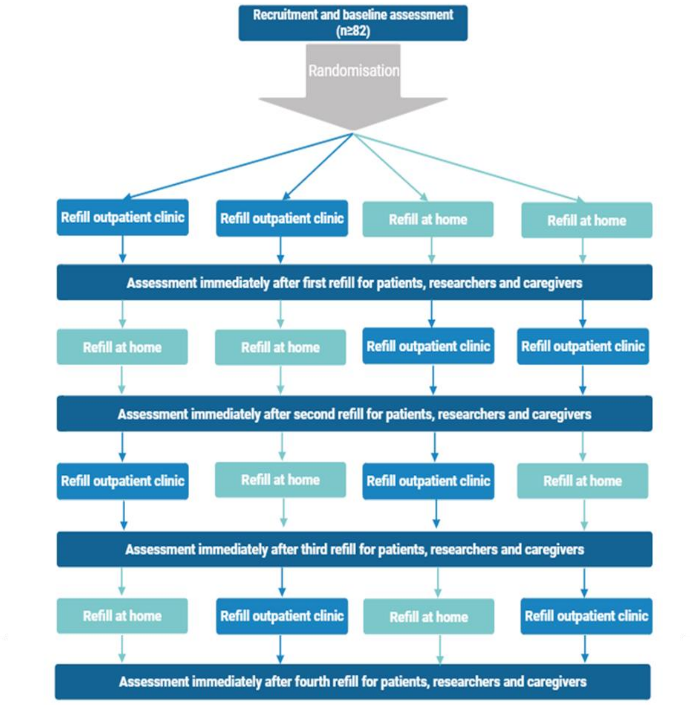

Fig. 1: *Study flowchart. Each patient will receive two refills in the outpatient clinic and two refills at home. Created in https://BioRender.com*

Detailed Study Assessments/Interventions

Experimental intervention: Hospital at Home (HAH)	
Description	The experimental intervention consists of two pump refills for each patient, administered at home. The procedure itself will remain unchanged; however, instead of receiving it at the hospital, the refills will be conducted in the patient's home environment. A researcher will visit each patient at their home; this will be performed under remote control.<br>In the hospital pharmacy of UZ Brussels, the day before the refill procedure, a sealed box will be prepared for every patient. This will be stored at room temperature. The prescription and medication label will be double-checked by the hospital pharmacist and the refill team.<br>Once the researcher arrives at the patient's home, the first step will be to set up the telemonitoring system. Both the researcher at home and the researcher in the hospital will log in to a secure web connection. The researcher at home wears smart glasses, enabling full remote visual and audio monitoring. The researcher at the hospital will have the same view as the researcher at the patient's home. The

	home researcher will then verbally confirm the pump settings and cross-check the medication label with the prior pump information. A final check is made when the researcher at the patients' home presents the medication label directly to the researcher in the hospital. After completing all verifications, the sterile refill procedure will be carried out again under remote supervision. The researcher at home will be a predoctoral neurosurgical resident, the researcher at the hospital will be a predoctoral researcher schooled in biomedical sciences.<br>Following the refill, a post-refill evaluation using an ultrasound device (Clarius C3 Scanner, Vancouver, Canada) will be performed to confirm that the pump was correctly refilled and that no subcutaneous drug injection occurred. Once this confirmation is secured, the remote connection will be terminated (Fig. 2, section 15). If the procedure cannot be carried out in a clean and sterile manner at the patient's home (e.g., due to poor hygiene or inadequate general body care), the home refill attempt will be considered unsuccessful. In that case, the procedure will be rescheduled and performed at the outpatient clinic within the same week. After the refill procedure, we suggest the presence of a caregiver during approximately 12 hours.
Smart glasses and telemonito-ring	To be able to perform refills at home in a safe way, we will test the feasibility of telemonitoring through smart glasses for this procedure. We aim to set up a communication channel between two people: the researcher performing the procedure at home, and another researcher at the VUB. Other practical details are described in the section just above.<br><br>The Real Wear Navigator 520 will be used to establish this channel. This piece of technology creates an augmented reality, in which high-yield visual and auditive connection can be established. The RealWear Navigator 520 is GDPR-compliant, both Bluetooth and Wi-Fi can be used to connect the device with an external internet source of user's choice. We will create a secure end-to-end connection through Microsoft Teams. The obtained connection will be a solely live connection, none of the visual nor auditive data will be stored.
Assessment	Saliva samples will be obtained with synthetic salivettes (Sarstedt AG & Co., Nümbrecht, Germany) at three different time points: five minutes before the refill, just after the refill and ten minutes after the refill.<br>Immediately after the termination of the teleconnection, the patient will receive a tablet with a link to the relevant questionnaires in the data collection tool. The test order will be randomized, but all will include: GCQ, MQOL, EQ-5D-5L, VAS, PROMIS, STAI, Likert-scale for patient satisfaction, GSE and questions about safety. Additionally, caregiver burden will be assessed using the ZBI. The quality of telemonitoring, duration of the consultation, and safety will be monitored by the researchers (at home and at the hospital).<br><br>All the above-mentioned data is study-specific, assessment does not occur during routine practice.
Intervention team	The intervention team consists of two main people: the researcher in the hospital and the researcher at home. The last one is responsible for the refill intervention. This person will go to the patient's home and perform the procedure.<br>The researcher, who will be at VUB/UZB, is the connection and will remain in close contact with the researcher during the refill at home.<br>This team will be supervised by the PI, Prof. dr. M. Moens and indirectly supervised by the outpatient, treating physician of the patient.

Control intervention	
Description	The control intervention consists of standard care. Five minutes before the refill, a saliva sample will be obtained. These synthetic salivettes will be provided by the researchers in each participating center.<br>The refill procedure will be carried out by the physician, following the standard care protocols currently in place at each participating hospital. During the visit, the pump settings are first read out, after which the sterile refill procedure is performed. The intrathecal pump is then refilled with the medication prescribed for the individual patient, either morphine, baclofen, adjuvantia or a combination of them.
Assessment	After the consultation, the patients will receive a tablet with the relevant questionnaires in the data collection tool. The test order will be randomized, but all will include: GCQ, MQOL, EQ-5D-5L, VAS, PROMIS, STAI, GSE, Likert-scale for patient satisfaction and questions about safety. Additionally, caregiver burden will be assessed using the ZBI. The duration of the consultation, and safety will be monitored by both the researcher at home and the one at the hospital.<br><br>All the above-mentioned data is study-specific; assessment does not occur during routine practice.

Data Collection and Management

Monitoring

Adverse events and adverse reactions

The use of intrathecal drug delivery (IDD) systems does have inherent risks. This study aims to perform IDD refills at home instead of at the hospital. Nevertheless, from a technical point of view, the performance of IDD refills at home carries the same risk as performing them in a hospital setting.

Definitions and reporting

An adverse event is defined as an unexpected medical occurrence in a participant after exposure to a medicine or treatment, which is not necessarily caused by that medicine or treatment. An AE can therefore be any unfavorable and unintended sign (including an abnormal laboratory finding), symptom or disease temporally associated with the use of a product, whether or not considered related to the product. Any worsening (i.e., any clinically significant adverse change in the frequency or intensity of a pre-existing condition) should be considered an AE.

Adverse reactions are defined as all untoward and unintended responses to medicinal products related to any dose administered.

Reporting period

An AE possibly related to the refill and the total number of AEs will be systematically reported from baseline until the end of the study.

Intensity

During the study, the investigator will grade the intensity of any adverse event or reaction as follow:
- Mild: Symptoms do alter patient's normal functioning.
- Moderate: Symptoms produce some degree of impairment to function, but are hazardous, uncomfortable, or embarrassing to the patient.
- Severe: Symptoms hazardous to well-being, significant impairment of function or incapacitation.

Relationship to treatment

The steering committee will determine the relationship to the treatment:
- **Definitely related:** There is clear evidence to suggest a causal relationship, and other possible contributing factors can be ruled out. The clinical event, including an abnormal laboratory test result, occurs in a plausible time relationship to the study intervention and cannot be explained by concurrent disease or other drugs or chemicals.
- **Probably related:** There is evidence to suggest a causal relationship, and the influence of other factors is unlikely. The clinical event, including an abnormal laboratory test result, occurs within a reasonable time after administration of the study intervention, is unlikely to be attributed to concurrent disease or other drugs or chemicals.
- **Potentially related:** There is some evidence to suggest a causal relationship (e.g., the event occurred within a reasonable time after administration of the study intervention). However, other factors may have contributed to the event (e.g., the participant's clinical condition, other concomitant events). Although an AE may rate only as "possibly related" soon after discovery, it can be flagged as requiring more information and later be upgraded to "probably related" or "definitely related", as appropriate.
- **Unlikely to be related**: A clinical event, including an abnormal laboratory test result, whose temporal relationship to the study intervention makes a causal relationship improbable (e.g., the event did not occur within a reasonable time after administration of the study intervention) and in which other drugs or chemicals or underlying disease provides plausible explanations (e.g., the participant's clinical condition, other concomitant treatments).
- **Not related:** The AE is completely independent of the study intervention, and/or evidence exists that the event is definitely related to another etiology. There must be an alternative, definitive etiology documented by the clinician.

Serious adverse event or reaction

Definition

A serious adverse event or serious adverse reaction is defined as any untoward medical occurrence or response to a medicinal product or treatment that at any dose:
- Results in death
- Is life-threatening[a]
- Results in patient hospitalization
- Results in a persistent or significant disability/incapacity
- Results in congenital anomalies/birth defects
- Important adverse events that are not immediately life-threatening or do not result in death or hospitalization but may jeopardize the patient or may require intervention to prevent one of the outcomes listed.

[a]The term "life threatening" in the definition of SAE refers to an event in which the subject was at risk of death at the time of the event. It does not refer to an event which hypothetically might have caused death if it was more severe.

Immediate reporting

The percentage of patients reporting an SAE possibly related to the treatment and the total number of SAEs reported will be systematically recorded from baseline assessment until the end of the study. All SAEs will be reported to the Competent Authorities (CA) and Ethics Committee within 24 hours.

Suspected unexpected serious adverse reaction (SUSAR)

Definition

A SUSAR is defined as a serious adverse reaction that occurs in a clinical trial participant, which is assessed as being suspected as well as unexpected, and which is having a reasonable possibility of having a causal relationship with the study medication.

Reporting

All SUSARs must be reported to the ethics committee and health authority as per EU (European Union) and Belgian legislation. The reporting period for SUSARs if fatal or life-threatening is as soon but not later than 7 days after becoming aware. For SUSARs not fatal or non-life threatening the reporting period is 15 days after becoming aware. The sponsor will report to the Ethics Committee and Health Authorities.

Procedures for handling special situations

Overdose and pocket refill management

Following the refill, a post-refill evaluation using an ultrasound device (Clarius C3 Scanner, Vancouver, Canada) will be performed to confirm that the pump was correctly refilled and that no subcutaneous drug injection occurred. If a pocket refill or symptoms of overdose are detected, an emergency procedure will be initiated.

Morphine

Symptoms:
- Respiratory failure
- Profound drowsiness or unresponsiveness
- Seizures
- Coma or loss of consciousness
- Bradycardia

If any of these symptoms occur, immediate medical intervention is essential. 112 will be called, it will be ensured that the airway remains clear, followed by placing the patient in a safe position.

Baclofen

Symptoms:
- Hypotonia or flaccid paralysis
- Decreased level of consciousness of coma
- Respiratory depression
- Hypotension
- Nausea and vomiting
- Seizures

In the event of suspected intrathecal baclofen overdose, immediate medical intervention is critical. 112 will be called, it will be ensured that the airway remains clear, followed by placing the patient in a safe position.

Data Collection

An Electronic Data Capture system "REDCap (Research electronic Data Capture)" will be used for data collection. The system is validated and access to all levels will be granted/revoked by the sponsor representative. Trial data should be entered within a reasonable time after the subject attended the visit. Corrections/modifications will be automatically tracked by an audit trail detailing the date and time of the correction and the name of the person performing the correction. Data will be collected through online self-reported questionnaires. Participants will be provided with a tablet with a link to

the relevant questionnaires. For participants who are unable to complete the questionnaires online, or in cases where internet connectivity is limited, paper-based versions of the questionnaires will be available.

IC will be obtained from all participants before the initiation of the study. This consent constitutes the legal basis for the collection, processing, and management of personal data in compliance with applicable data protection regulations.

To support participant retention and ensure completion of study procedures, patients will receive a reminder of their upcoming refill appointment. Following each refill, they will be given a tablet with the relevant questionnaires.

Database Management and Quality Control

During the research, data management and storage will be overseen by the pre-doctoral investigators. All collected information will be saved on a dedicated, system-encrypted Pixiu or SharePoint site at Vrije Universiteit Brussel, with access restricted to the investigators and supervisors. A secure external hard drive will serve as an additional backup. Once the study concludes, responsibility for the data will transfer to the principal investigator, and all files will be migrated to the Vrije Universiteit Brussel Archive for a 25-year retention period. Prior to archiving, any direct personal identifiers will be permanently removed.

Personal data will be processed in accordance with the applicable EU regulations, including the General Data Protection Regulation (GDPR), the Belgian Law of 30 July 2018 on the protection of personal data, and in compliance with Good Clinical Practice (GCP) guidelines. Pseudonymization will be performed as soon as data collection starts. As an additional safety measure, the file linking the pseudonymization with the original direct identifiers will be encrypted before uploading it on Pixiu/SharePoint.

Specific measures will be implemented to prevent unauthorized access, as personally identifiable data will be collected. REDCap will be used for data collection. To enhance data protection, responses to the questionnaires will only be accessible to the study team. Collected data will be protected by a password. Additionally, personally identifiable information and clinical trial data will be stored separately, with the last one linked only to a unique participant ID. Access to ICs, personally identifiable data and the linkage between identifiers and participant ID will be restricted to the investigators and supervisors and will be stored separately from the clinical trial data.

Vrije Universiteit Brussel adheres to the FOSB metadata standard, developed by the Flemish Open Science Board, which is compatible with the international DataCite metadata schema. At the project level, essential metadata—including the project title, investigators, objectives, hypotheses, funding information, study protocol, sampling procedures, data collection instruments, and technical infrastructure (hardware and software)—will be documented and made available through research plans and publications. At the database level, an inventory of the data files will be provided via a comprehensive read-me file. At the data level, a codebook will accompany the dataset, outlining the structure and meaning of quantitative variables, along with the scripts used for data analysis.

Personal data will be processed in accordance with the EU's Data Protection Directive (Directive 95/46/EC) and regulation EC45/2001, the relevant Belgian legislation concerning data protection of July 30th, 2018, and GCP. As we collect personal identifiable data, the following steps are taken to limit unauthorized access. ICs will be preserved at a secure location at the VUB. REDCap will be used for data collection to improve data protection as responses to questionnaires will only be accessible to the study team. Collected data will be password protected. Personal identifiable and clinical trial data will be separated, with the latter receiving a unique participant ID. Access to ICs, personal identifiable data and the link with the participant ID will be restricted to the investigators and supervisors and stored separately from the trial data. Eventual further dissemination of data will only occur in a pseudonymized or aggregated way.

Statistical Considerations and Data Analysis

Baseline analysis

Data processing and statistical analysis will be performed on pseudonymized data, in a blinded way. No interim analysis will be performed.

Using baseline data, cross-sectional results will be provided on general comfort, QoL, pain intensity, pain interference, stress, anxiety, self-efficacy, and burden of caregivers for patients who receive IDD. In addition to describing this population, supervised and non-supervised machine learning techniques will be applied such as hierarchical clustering, mediation analysis and regression models to explore the relations between baseline variables.

Main analysis

To evaluate and compare therapy effects, mixed model analysis will be used. The need for random intercepts and slopes will be assessed. Potential confounding variables, such as baseline stress and anxiety levels, will be taken into account in the analysis. Statistical as well as clinical significance differences will be defined at $\alpha < 0.05$. Additionally, based on baseline data, predictive modelling will be conducted to identify which patients will benefit the most from home-based refill procedures. This will involve the application of both supervised and unsupervised machine learning techniques (K-means clustering, decision trees, and Random Forest algorithms). All statistical analysis will be performed in SAS, SPSS and R.

Health economic analysis

A within-trial health economic evaluation will be conducted to assess the cost-effectiveness of home-based intrathecal pump refills compared to standard outpatient clinic refills. The analysis will include all participants, covering the period up to just before the third refill procedure. Each participant will receive one refill at home and one in the outpatient clinic, with a follow-up period of approximately 2–3 months between the refills.
The analysis will follow the intention-to-treat principle (79). Data on resource use will be collected through telephone interviews, as described in the outcome section ‘health expenditure’. The valuation of resource use will be based on official Belgian national tariffs.

Health outcomes will be assessed in two ways:

1. Percentage increase in comfort, as measured by the General Comfort Questionnaire, is the trial’s primary outcome.
2. Utility values, derived from EQ-5D questionnaires and converted using Belgian population-based value sets, in line with national health economic guidelines (55).

The outpatient refill procedure will serve as the control group. Any missing data will be addressed before analysis using appropriate methods for handling incomplete health economic data (79).
Cost differences between the two groups will be analyzed using generalized linear models. The Modified Park Test will guide the selection of the appropriate model specification (80). The main result will be the Incremental Cost-Effectiveness Ratio (ICER) (81), calculated with the estimation:

$$ICER = \frac{(incremental\ cost)}{(percentage\ increment\ in\ functioning)}$$

and

$$ICER = \frac{(incremental\ cost)}{(incremental\ QALY\ gained)}$$

To account for uncertainty, probabilistic sensitivity analysis will be conducted (79). Non-parametric bootstrapping will be used to assess variability in cost and outcome estimates. These results will be illustrated using Cost-Effectiveness Acceptability Curves (CEACs), showing the probability that the intervention is cost-effective at various willingness-to-pay (WTP) thresholds. All results will be reported in accordance with the Consolidated Health Economic Evaluation Reporting Standards (CHEERS) guidelines (82).

In addition to the within-trial economic evaluation, a model-based analysis will be conducted to estimate the long-term costs and health outcomes of the intervention (home-based refills) versus the control (outpatient refills) beyond the trial follow-up period.

A Markov model will be developed in accordance with established health economic modelling guidelines. The model will use a one-year cycle length and a lifetime horizon to capture all relevant long-term effects. Lifetime incremental costs and QALY's will be calculated for both the home-based and outpatient refill procedures. Discount rates of 3% for costs and 1.5% for utilities will be applied, this is in line with the Belgian health economic guidelines (83,84).

The model will include probabilistic sensitivity analysis to account for parameter uncertainty, following the same approach as the within-trials evaluation. Again, results will be visualized using CEACs to illustrate the probability that the home-based intervention is cost-effective at various WTP thresholds. All modeling results will be reported in line with the CHEERS reporting standards (82).

<u>Willingness-to-pay (WTP)</u>

In addition to the cost-utility analysis, where QoL and life-years play a prominent role, a DCE will be conducted to estimate the WTP for pump refills at home. This preference elicitation method will help inform future reimbursement policy decisions by capturing the broader value that society places on the intervention (85).

A DCE is based on the idea that people's choices between different options depend on specific characteristics, or attributes, of those options. By systematically varying these attributes across scenarios and asking participants to choose between them, it is possible to estimate how much each attribute influences decision-making. This method will allow reverse engineer choice to quantify the impact of changes in attribute levels on choice. The results will complement the health economic evaluation by providing insight into preferences and perceived value from a societal perspective (86).

A literature review has been conducted to obtain the variables that represent the patient-centered values relevant to DCE (87–97). The following attributes will be used:

- Travel time and waiting time
- Person who performs the refill procedure
- Risk of wound infection
- Additional required amount that you should have to pay (non-reimbursed)

Each attribute is divided into multiple levels (Table 2, section 15). By combining different attributes and their corresponding levels, various scenarios can be created. These attributes will be presented in a choice set consisting of two scenarios (A and B), each representing different combinations of attribute levels (Table 2, section 15). Patients will be asked to choose between the two scenarios presented. During the study, patients will be asked to complete the DCE after the fourth refill. An example of such a choice set is shown in Table 3 (section 15).

Ethical Considerations

Ethical Conduct of the Study

Declaration of Helsinki

The trial will be performed in accordance with the Declaration of Helsinki, the conditions and principles of GCP, the protocol and applicable local regulatory requirements and laws.

Ethics Committee (EC)

Before the start of the trial or implementation of any amendment, approval of the trial protocol and amendments, ICFs and other relevant documents will be obtained from the applicable ethical committee.
An annual progress report will be submitted by the sponsor to the central EC within 30 days of the anniversary date on which the favorable opinion was given, and annually until the trial is declared ended.

Within one year after the end of the study, the investigator will submit a final clinical study report with the results, including any publications/abstracts, to the Sponsor, which in turn submits it to the central EC. The PI will notify the sponsor of the end of the study and the sponsor will notify the central EC of the end of the study. If the study is ended prematurely, the investigator will notify the central EC, including the reasons for the premature termination. All correspondence with the EC will be retained in the TMF/ISF.

Recruitment and Informed Consent (IC)

Eligible patients will only be included in the study after providing a written IC. IC will be obtained prior to conducting any study-specific procedures (as described in this protocol). These ICs will be collected on paper and then digitized, after which the paper versions will be destroyed.
Before enrolling in the study, the investigator will explain the study and the implications of participation to potential participants. Participants will be informed that their participation is voluntary and that they may withdraw consent to participate at any time. Participants will be told that their records may be accessed by CA and by authorized persons without violating the confidentiality of the participant, to the extent permitted by the applicable law(s) and/or regulations. By signing the ICF, the participant is authorizing such access.

After this explanation and before entry to the study, written, dated, and signed IC will be obtained from the participant. The ICF will be provided in a language sufficiently understood by the participant. Participants are given the opportunity to ask questions. The participant will be given sufficient time to read the ICF and to ask additional questions. After this explanation and before entry to the study, consent will be appropriately recorded by means of the participant's dated signature. After having obtained the consent, a copy of the ICF is given to the participant.

Sample size calculation

The sample size calculation (G*Power version 3.1.9.4) is performed on patient comfort, measured with the general comfort questionnaire, as primary efficacy outcome variable after 4 intrathecal pump refill procedures. True mean comfort responses are based on the study of Zhao et al. (2021) (98), and a common standard deviation is calculated based on three studies which resulted in a value of 16.01049 (98–100). This results in an effect size of f=0.3035623. To conduct a randomized clinical trial with 2 intrathecal pump refill procedures at home and 2 procedures in the outpatient clinic with estimated true mean comfort responses for the outpatient clinic refill of 78.47 and 88.78 for the refill at home, with a common standard deviation of 16.01049, and 85% power to detect differences in means at a 5% two-sided significance level, a total sample size of 82 patients is needed. This sample size considers a 20% loss to follow-up after 4 refill procedures.

Study Data Protection

The collection and processing of personal data from participants enrolled in the study will be limited to those data that are necessary to fulfill the objectives of this study. These data must be collected and processed with adequate precautions to ensure confidentiality and compliance with applicable data protection laws and regulations.

An eCRF will be available in REDCap. The eCRF will be completed for all participating patients. This eCRF will include specific pages for inclusion and exclusion criteria, and for reporting each visit. Other specific pages will be dedicated to concomitant treatments and AEs (non-serious and serious). The investigator will review, approve, and validate each completed eCRF; the investigator's signature (validation) serving as attestation of the investigator's responsibility for ensuring that all data entered on the eCRF are complete, accurate and authentic. All data will be processed according to the principles that the new European General Data Protection Regulation (GDPR) imposes, which are in force since 25 May 2018. The STIMULUS Research Group, Vrije Universiteit Brussel, will be responsible for the processing of personal data. The Data Protection officer is dpo@vub.be. The processing of personal data is for scientific research purposes and will happen on the legal basis of consent that can be withdrawn by the patient. All researchers involved in this clinical trial or in research projects that use materials original from this clinical trial are potential recipients of the personal data, as well as staff involved in monitoring and ethical evaluation and people from CA and subcontracted parties that perform analysis on study-related data or materials. It is possible that personal data will be viewed by people who are in countries that do not use the same standards as the EU in terms of legal protection of data. In that case, we guarantee that the conditions of European and Belgian legislation on the protection of personal data will be respected. The study-related documents will be stored for at least 25 years.

Subject Identification

The participant identification will be treated as confidential and will be filed by the investigator in an identification log. This log is kept at the participating site and shall not be copied. In all reports and communication between the site and the Sponsor the participant shall be identified with a participant study number.

Insurance

VUB is, as sponsor of the trial, responsible for ensuring appropriate general/product liability insurance and as required in accordance with applicable laws and regulations, country-specific liability insurance coverage for claims made by a trial subjects for injury arising from the subject's participation in the trial.
In accordance with the Belgian law relating to experiments in humans dated May 7, 2004 the sponsor shall be liable, even without fault for any damages incurred by the study participant and linked directly or indirectly to the participation to the study and the sponsor shall provide compensation therefore through its insurance program. Before commencing the study, the sponsor shall enter an insurance contract which covers this liability, and the liability of every individual intervening in the study, irrespective of the nature of the affiliation between the intervening individual, the sponsor and the study participant. Every contractual provision aiming at limiting this liability is considered null and void.

The participating site, the investigator and sponsor shall have and maintain in full force and effect during the term of this Agreement (and following termination or completion of the Study to cover any claims arising from the Study) adequate insurance coverage for: (i) medical professional and/or medical malpractice liability, and (ii) general liability resulting from the study at the participating site required by local law, each such insurance coverage in amounts appropriate to the conduct of the services of the participating site, the investigator and sponsor under this agreement. The participating site and sponsor shall be solely responsible for any deductible or self-insured retention under any such policies.

Reporting and Dissemination

The data and information collected during this trial will be reported in a publication in a scientific/medical journal. Reporting of trial results will be performed according to local regulations.

For the correct authorship rules we refer to the International Committee of Medical Journal Editors: https://www.icmje.org/recommendations/browse/roles-and-responsibilities/defining-the-role-of-authors-and-contributors.html

Finance and Conflict of Interest Statement

This study is funded by Research Foundation Flanders through the TBM program (project number T000525N). Investigators and study team members will provide the sponsor with sufficient, accurate financial information in accordance with local regulations to allow the sponsor to submit complete and accurate financial certification or disclosure statements to the appropriate regulatory authorities/ethics committee. Any update of information on financial interests should be disclosed during the course of the study.

Tables and Figures

What?	Patient comfort *	QoL *	Pain Intensity *	Pain interference *	Stress *	Anxiety *	Self-efficacy *	Burden caregivers *	Patient satisfaction	Tele-monitoring quality** + time	Safety	Health expenditure ***	Patient preference
How?	GCQ	MQOL + EQ-5D	VAS	PROMIS	Sarstedt AG & Co	STAI	GSE	ZBI	Likert-scales	Likert-scales + time expenditure	Overall safety + environmental safety + sterile procedure	Phone calls	Multiple choice + DCE
When?	At baseline * and after each intrathecal pump refill (** only applicable for refills at home)<br>***Four weeks after first refill and four weeks after second refill												After last refill
Who?	Patient	Patient	Patient	Patient	Salivary sample	Patient	Patient	Caregiver	Patient	Researchers	Patient + researchers	Patient	Patient

Table 1: *Overview outcomes. Abbreviations: GCQ = General Comfort Questionnaire; MQOL =* McGill Quality of Life Questionnaire; EQ-5D = EuroQol with five dimensions; VAS = Visual Analogue Scale; PROMIS = Patient-Reported Outcomes Measurement Information System; STAI = State Trait Anxiety Inventory; GSE = General Self-Efficacy; ZBI = Zarit Burden Interview, DCE = Discrete choice experiment.

Attributes	Levels
Travel time + waiting time	• 0 minutes<br>• 45 minutes<br>• 90 minutes
Person who performs the refill	• Always the same person<br>• Team of 2 persons (alternating)<br>• Always different
Risk of wound infection	• 1 in 100 refills<br>• 1 in 500 refills<br>• 1 in 1.000 refills
Additional required amount that you should have to pay (non-reimbursed)	• 0 euro<br>• 40 euros<br>• 70 euros

Table 2: *Varying levels of different attributes.*

	Scenario A (refill at home)	Scenario B (refill at the hospital)
**Travel time + waiting time**	There is no extra travel time or waiting time	Your total travel and waiting time is 90 minutes.
**Person who performs the refill**	Every refill is performed by a different person	Every refill is performed by the same person
**Risk of wound infection**	The risk of a wound infection for you is 1 in 500 refills	The risk of a wound infection for you is 1 in 1.000 refills
**Additional required amount that you should have to pay (non-reimbursed)**	The cost for you is 40 euros.	The cost for you is 0 euros

Table 3: *Example of a choice set "Which scenario do you prefer for your refill procedure?".*

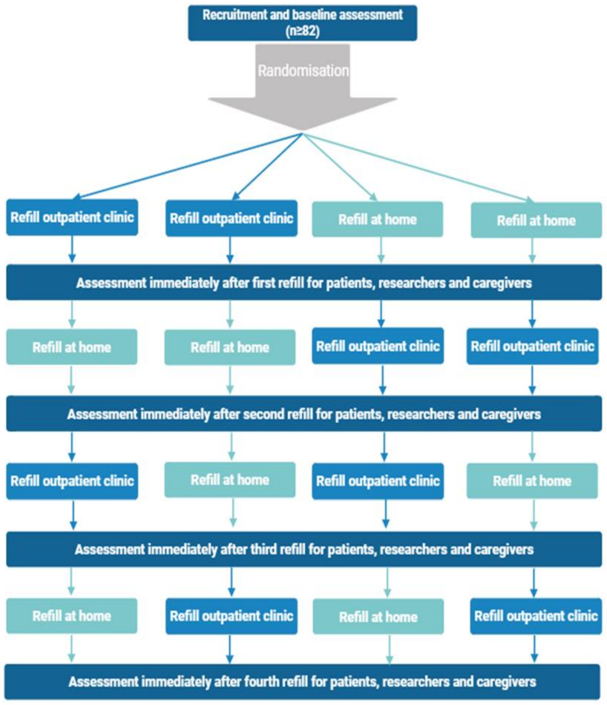

Fig. 1: *Study flowchart. Created in https://BioRender.com*

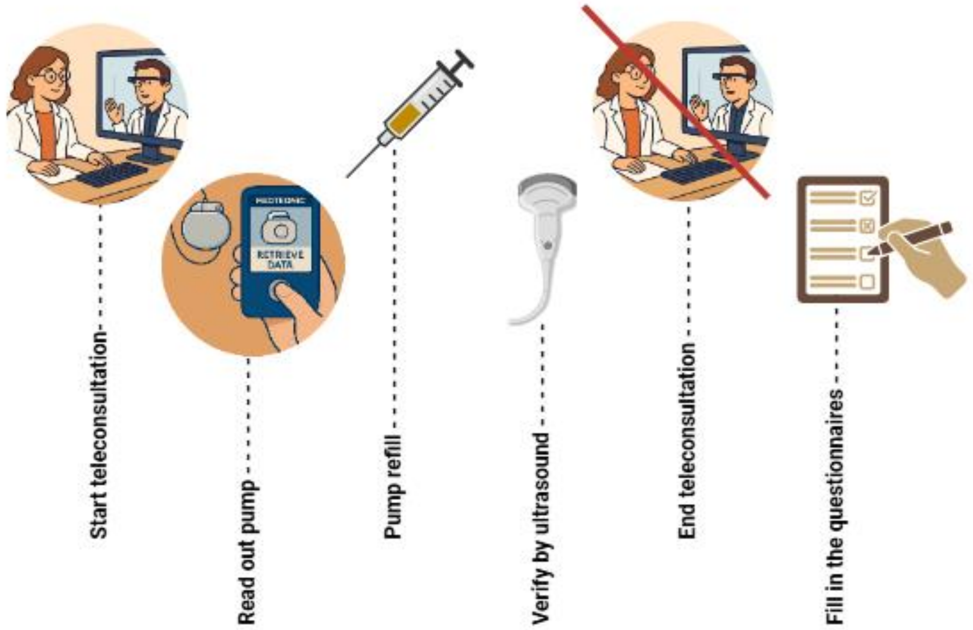

Fig. 2: *Overview home visit. Created in* [*https://BioRender.com*](https://BioRender.com)

References

1. Nicholas MK, Costa DSJ, Blanchard M, Tardif H, Asghari A, Blyth FM. Normative data for common pain measures in chronic pain clinic populations: Closing a gap for clinicians and researchers. Pain. 2019 May 1;160(5):1156–65.
2. Treede RD, Rief W, Barke A, Aziz Q, Bennett MI, Benoliel R, et al. Chronic pain as a symptom or a disease: The IASP Classification of Chronic Pain for the International Classification of Diseases (ICD-11). Vol. 160, Pain. Lippincott Williams and Wilkins; 2019. p. 19–27.
3. Johannes CB, Le TK, Zhou X, Johnston JA, Dworkin RH. The Prevalence of Chronic Pain in United States Adults: Results of an Internet-Based Survey. Journal of Pain. 2010 Nov;11(11):1230–9.
4. Fayaz A, Croft P, Langford RM, Donaldson LJ, Jones GT. Prevalence of chronic pain in the UK: a systematic review and meta-analysis of population studies. Open [Internet]. 2016;6:10364. Available from: http://dx.doi.org/
5. Yong RJ, Mullins PM, Bhattacharyya N. Prevalence of chronic pain among adults in the United States. Pain. 2022 Feb 1;163(2):E328–32.
6. Thomson S, Jacques L. Demographic characteristics of patients with severe neuropathic pain secondary to failed back surgery syndrome. Pain Practice. 2009;9(3):206–15.
7. Mirzaei M, Rahmaninan M, Mirzaei M, Nadjarzadeh A, Dehghani Tafti AA. Epidemiology of diabetes mellitus, pre-diabetes, undiagnosed and uncontrolled diabetes in Central Iran: Results from Yazd health study. BMC Public Health. 2020 Feb 3;20(1).
8. Majlesi J. Patients with Chronic Musculoskeletal Pain of 3–6-Month Duration Already Have Low Levels of Health-Related Quality of Life and Physical Activity. Curr Pain Headache Rep. 2019 Nov 1;23(11).
9. Jonas WB, Crawford C, Colloca L, Kriston L, Linde K, Moseley B, et al. Are Invasive Procedures Effective for Chronic Pain? A Systematic Review. Pain Medicine (United States). 2019 Jul 1;20(7):1281–93.
10. Busse JW, Wang L, Kamaleldin M, Craigie S, Riva JJ, Montoya L, et al. Opioids for Chronic Noncancer Pain: A Systematic Review and Meta-analysis. Vol. 320, JAMA - Journal of the American Medical Association. American Medical Association; 2018. p. 2448–60.

11. Dowell D, Haegerich TM. Using the CDC Guideline and Tools for Opioid Prescribing in Patients with Chronic Pain HHS Public Access [Internet]. Vol. 93, Am Fam Physician. 2016. Available from: http://www.cdc.gov/drugoverdose/prescribing/resources.html.
12. Philpot LM, Ramar P, Elrashidi MY, Mwangi R, North F, Ebbert JO. Controlled Substance Agreements for Opioids in a Primary Care Practice. J Pharm Policy Pract. 2017 Sep 12;10(1).
13. Al-Kaisy A, Van Buyten JP, Carganillo R, Caraway D, Gliner B, Subbaroyan J, et al. 10 kHz SCS therapy for chronic pain, effects on opioid usage: Post hoc analysis of data from two prospective studies. Sci Rep. 2019 Dec 1;9(1).
14. Organisation for Economic Co-operation and Development. Health at a Glance 2019: OECD Indicators [Internet]. Paris; 2019 [cited 2025 Jun 11]. Available from: https://www.oecd.org/en/publications/health-at-a-glance-2019_4dd50c09-en.html
15. Noori A, Sadeghirad B, Wang L, Siemieniuk RAC, Shokoohi M, Kum E, et al. Comparative benefits and harms of individual opioids for chronic non-cancer pain: a systematic review and network meta-analysis of randomised trials. Vol. 129, British Journal of Anaesthesia. Elsevier Ltd; 2022. p. 394–406.
16. Chou R, Turner JA, Devine EB, Hansen RN, Sullivan SD, Blazina I, et al. The effectiveness and risks of long-term opioid therapy for chronic pain: A systematic review for a national institutes of health pathways to prevention workshop. Vol. 162, Annals of Internal Medicine. American College of Physicians; 2015. p. 276–86.
17. Duarte R, Copley S, Nevitt S, Maden M, Al-Ali AM, Dupoiron D, et al. Effectiveness and Safety of Intrathecal Drug Delivery Systems for the Management of Cancer Pain: A Systematic Review and Meta-Analysis. Vol. 26, Neuromodulation. International Neuromodulation Society; 2023. p. 1126–41.
18. Capozza MA, Triarico S, Mastrangelo S, Attinà G, Maurizi P, Ruggiero A. Narrative review of intrathecal drug delivery (IDD): indications, devices and potential complications. Ann Transl Med. 2021 Jan;9(2):186–186.
19. Bottros MM, Christo PJ. Current perspectives on intrathecal drug delivery. Vol. 7, Journal of Pain Research. Dove Medical Press Ltd.; 2014. p. 615–26.
20. Miyake MM, Bleier BS. The blood-brain barrier and nasal drug delivery to the central nervous system. Am J Rhinol Allergy. 2015 Mar 1;29(2):124–7.
21. Perruchoud C, Dupoiron D, Papi B, Calabrese A, Brogan SE. Management of Cancer-Related Pain With Intrathecal Drug Delivery: A Systematic Review and Meta-Analysis of Clinical Studies. Vol. 26, Neuromodulation. International Neuromodulation Society; 2023. p. 1142–52.
22. Marathe A, Allahabadi S, Abd-Elsayed A, Saulino M, Hagedorn JM, Orhurhu V, et al. Intrathecal Baclofen Monotherapy and Polyanalgesia for Treating Chronic Pain in Patients with Severe Spasticity. Vol. 25, Current Pain and Headache Reports. Springer; 2021.
23. Kadakia KT, Balatbat CA, Siu AL, Cohen IG, Wilkins CH, Dzau VJ, et al. Hospital-at-Home: Multistakeholder Considerations for Program Dissemination and Scale. Vol. 100, Milbank Quarterly. John Wiley and Sons Inc; 2022. p. 673–701.
24. Paulson MR, Torres-Guzman RA, Avila FR, Maita KC, Forte AJ, Butera JE, et al. Telemedicine allows for effective communication between a medically complex cancer patient and his virtual hospital medical team. Clin Case Rep. 2022 Oct;10(10).
25. Wujcik D, Dudley WN, Dudley M, Gupta V, Brant J. Electronic Patient Symptom Management Program to Support Patients Receiving Cancer Treatment at Home During the COVID-19 Pandemic [Internet]. Available from: www.elsevier.com/locate/jval
26. Nipp RD, Shulman E, Smith M, Brown PMC, Johnson PC, Gaufberg E, et al. Supportive oncology care at home interventions: protocols for clinical trials to shift the paradigm of care for patients with cancer. BMC Cancer. 2022 Dec 1;22(1).
27. Leonardsen ACL, Helgesen AK, Stensvold A, Magnussen J, Grøndahl VA. Cancer patients' perspectives on remote monitoring at home during the COVID-19 pandemic- a qualitative study in Norway. BMC Health Serv Res. 2022 Dec 1;22(1).
28. Qaddoura A, Yazdan-Ashoori P, Kabali C, Thabane L, Haynes RB, Connolly SJ, et al. Efficacy of hospital at home in patients with heart failure: A systematic review and meta-analysis. PLoS One. 2015 Jun 8;10(6).
29. Shepperd S, Gonçalves-Bradley DC, Straus SE, Wee B. Hospital at home: home-based end-of-life care. Vol. 2021, Cochrane Database of Systematic Reviews. John Wiley and Sons Ltd; 2021.

30. Tibaldi V, Isaia G, Scarafiotti C, Gariglio F, Zanocchi M, Bo M, et al. HEALTH CARE REFORM Hospital at Home for Elderly Patients With Acute Decompensation of Chronic Heart Failure A Prospective Randomized Controlled Trial.
31. Patel H, Shafazand M, Ekman I, Höjgård S, Swedberg K, Schaufelberger M. Home care as an option in worsening chronic heart failure- A pilot study to evaluate feasibility, quality adjusted life years and cost-effectiveness. Eur J Heart Fail. 2008 Jul;10(7):675–81.
32. Gofeld M, McQueen CK. Ultrasound-Guided Intrathecal Pump Access and Prevention of the Pocket Fillp me_1090 607..611 [Internet]. Available from: https://academic.oup.com/painmedicine/article/12/4/607/1869241
33. Patel AK, Dowling M, Purcell A, O'Brien J, Moore DM. Managing a National Intrathecal Pump Service During the COVID-19 Pandemic. Neuromodulation. 2020 Oct 1;23(7):922–5.
34. Coffey RJ, Ridgely PL. Abrupt Intrathecal Baclofen Withdrawal: Management of Potentially Life-Threatening Sequelae. 2001.
35. Shanthanna H, Strand NH, Provenzano DA, Lobo CA, Eldabe S, Bhatia A, et al. Caring for patients with pain during the COVID-19 pandemic: consensus recommendations from an international expert panel. Vol. 75, Anaesthesia. Blackwell Publishing Ltd; 2020. p. 935–44.
36. Goudman L, De Smedt A, Huygens R, Noppen M, Vanschoenwinkel M, Hatem SM, et al. Hospital at home for intrathecal pump refills: A prospective effectiveness, safety and feasibility study. J Clin Med. 2021 Nov 1;10(22).
37. Goslinga-van der Gaag SME, Delhaas EM, Frankema SPG, Huygen FJPM. Efficiency and Safety of Aftercare With Intrathecal Baclofen on Location. Neuromodulation. 2019 Oct 1;22(7):828–33.
38. van der Gaag SME, Frankema SPG, van der Ploeg ES, Baart SJ, Huygen FJMP. Evaluating Community-Based Intrathecal Baclofen Therapy: Effectiveness, Safety, and Feasibility. J Clin Med. 2024 Apr 1;13(7).
39. Gonçalves-Bradley DC, Iliffe S, Doll HA, Broad J, Gladman J, Langhorne P, et al. Early discharge hospital at home. Vol. 2017, Cochrane Database of Systematic Reviews. John Wiley and Sons Ltd; 2017.
40. Verjan CR, Augusto V, Xie X, Buthion V. Economic comparison between Hospital at Home and traditional hospitalization using a simulation-based approach. Vol. 26, Journal of Enterprise Information Management. 2013. p. 135–53.
41. Mitrasinovic S, Camacho E, Trivedi N, Logan J, Campbell C, Zilinyi R, et al. Clinical and surgical applications of smart glasses. Vol. 23, Technology and Health Care. IOS Press; 2015. p. 381–401.
42. Munusamy T, Karuppiah R, Bahuri NFA, Sockalingam S, Cham CY, Waran V. Telemedicine via Smart Glasses in Critical Care of the Neurosurgical Patient—COVID-19 Pandemic Preparedness and Response in Neurosurgery. World Neurosurg. 2021 Jan 1;145:e53–60.
43. Barba P, Stramiello J, Funk EK, Richter F, Yip MC, Orosco RK. Remote telesurgery in humans: a systematic review. Vol. 36, Surgical Endoscopy. Springer; 2022. p. 2771–7.
44. Delhaas EM, Beersen N, Ken Redekop W, Klazinga NS. Long-Term Outcomes of Continuous Intrathecal Baclofen Infusion for Treatment of Spasticity: A Prospective Multicenter Follow-Up Study [Internet]. Vol. 11. 2008. Available from: http://www.blackwell-synergy.com/loi/ner
45. Stearns LM, Abd-Elsayed A, Perruchoud C, Spencer R, Hammond K, Stromberg K, et al. Intrathecal Drug Delivery Systems for Cancer Pain: An Analysis of a Prospective, Multicenter Product Surveillance Registry. Anesth Analg. 2020 Feb 1;130(2):289–97.
46. Perruchoud C, Eldabe S, Durrer A, Bovy M, Brookes M, Madzinga G, et al. Effects of Flow Rate Modifications on Reported Analgesia and Quality of Life in Chronic Pain Patients Treated with Continuous Intrathecal Drug Therapyp me_1088 571..576 [Internet]. Available from: https://academic.oup.com/painmedicine/article/12/4/571/1868516
47. Biggs SA, Duarte R V., Raphael JH, Ashford RL. Influence of a latent period in QALY anaylsis: Pilot study of intrathecal drug delivery systems for chronic non-malignant pain. Br J Neurosurg. 2011 Jun 28;25(3):401–6.
48. Kumar K, Bodani V, Bishop S, Tracey S. Use of intrathecal bupivacaine in refractory chronic nonmalignant pain. Vol. 10, Pain Medicine. 2009. p. 819–28.
49. Kumar K, Rizvi S, Bishop S. Cost Effectiveness of Intrathecal Drug Therapy in Management of Chronic Nonmalignant Pain. Clin J Pain. 2013 Feb;29(2):138–45.
50. Gonzalez-Baz MD, Pacheco del Cerro E, Ferrer-Ferrándiz E, Araque-Criado I, Merchán-Arjona R, de la Rubia Gonzalez T, et al. Psychometric validation of the Kolcaba General Comfort Questionnaire in critically ill patients. Australian Critical Care. 2023 Nov 1;36(6):1025–34.

51. Kolcaba K. Comfort Theory and Practice. 2003. 14–14 p.
52. Kolcaba K. Comfort Theory and Practice. 2003. 62–62 p.
53. T. De Vrieze, D. Coeck, H. Verbelen, N. Devoogdt, W. Tjalma, N. Gebruers. Cross-cultural Psychometric Evaluation of the Dutch McGillQoL Questionnaire for Breast Cancer Patients. 2016;
54. Cohen R, Fellow R, Mcgill M, Hospital RV. The McGill Quality of Life Questionnaire: a measure of quality of life appropriate for people with advanced disease. A preliminary study of validity and acceptability.
55. Bouckaert N, Cleemput I, Devriese S, Gerkens S. An EQ-5D-5L Value Set for Belgium. Pharmacoecon Open. 2022 Nov 1;6(6):823–36.
56. Ogon M, Krismer M, S611ner W, Kantner-Rumplmair W, Lampe A. Chronic low back pain measurement with visual analogue scales in different settings. Vol. 64, Pain. 1996.
57. Jensen MP, Karoly P, Braver S. The measurement of clinical pain intensity: a comparison of six methods. Pain. 1986;27(1):117–26.
58. Alghadir AH, Anwer S, Iqbal A, Iqbal ZA. Test-retest reliability, validity, and minimum detectable change of visual analog, numerical rating, and verbal rating scales for measurement of osteoarthritic knee pain. J Pain Res. 2018 Apr 26;11:851–6.
59. Amtmann D, Cook KF, Jensen MP, Chen WH, Choi S, Revicki D, et al. Development of a PROMIS item bank to measure pain interference. Pain. 2010 Jul;150(1):173–82.
60. Haws BE, Khechen B, Bawa MS, Patel D V., Bawa HS, Bohl DD, et al. The Patient-Reported Outcomes Measurement Information System in spine surgery: A systematic review. J Neurosurg Spine. 2019 Mar 1;30(3):405–13.
61. Turner AI, Smyth N, Hall SJ, Torres SJ, Hussein M, Jayasinghe SU, et al. Psychological stress reactivity and future health and disease outcomes: A systematic review of prospective evidence. Vol. 114, Psychoneuroendocrinology. Elsevier Ltd; 2020.
62. Chrousos GP. Stress and disorders of the stress system. Vol. 5, Nature Reviews Endocrinology. 2009. p. 374–81.
63. Skoluda N, La Marca R, Gollwitzer M, Müller A, Limm H, Marten-Mittag B, et al. Long-term stability of diurnal salivary cortisol and alpha-amylase secretion patterns. Physiol Behav. 2017 Jun 1;175:1–8.
64. Farzi A, Fröhlich EE, Holzer P. Gut Microbiota and the Neuroendocrine System. Vol. 15, Neurotherapeutics. Springer New York LLC; 2018. p. 5–22.
65. Şemsi R, Kökbaş U, Arslan B, Ergünol E, Kayrın L, Sepici Dinçel A. The Saliva Cortisol and Amylase Levels Related with Stress Response Compared by Different Analytical Methods. Appl Biochem Biotechnol. 2022 Mar 1;194(3):1166–77.
66. Richard L, Metzger. A reliablity and validity study of the state-trait anxiety inventory. J Clin Psychol. 1976;
67. Miles A, McManus C, Feinmann C, Glover L, Harrison S, Pearce S. The factor structure of the BDI in facial pain and other chronic pain patients: A comparison of two models using confirmatory factor analysis. Br J Health Psychol. 2001 May;6(2):179–96.
68. Samulowitz A, Hensing G, Haukenes I, Bergman S, Grimby-Ekman A. General self-efficacy and social support in men and women with pain – irregular sex patterns of cross-sectional and longitudinal associations in a general population sample. BMC Musculoskelet Disord. 2022 Dec 1;23(1).
69. Hott A, Pripp AH, Juel NG, Liavaag S, Brox JI. Self-efficacy and Emotional Distress in a Cohort With Patellofemoral Pain. Orthop J Sports Med. 2022 Mar 8;10(3).
70. Nicholas MK. The pain self-efficacy questionnaire: Taking pain into account. European Journal of Pain. 2007 Feb;11(2):153–63.
71. Lacasse A, Bourgault P, Tousignant-Laflamme Y, Courtemanche-Harel R, Choinière M. Development and validation of the French-canadian chronic pain self-efficacy scale [Internet]. Vol. 20, Pain Res Manag. 2015. Available from: http://patienteducation.stanford.edu/research/
72. Rashid M, Kristofferzon ML, Heiden M, Nilsson A. Factors related to work ability and well-being among women on sick leave due to long-term pain in the neck/shoulders and/or back: A cross-sectional study. BMC Public Health. 2018 May 30;18(1).
73. Luszczynska A, Scholz U, Schwarzer R. The General Self-Efficacy Scale: Multicultural Validation Studies. J Psychol. 2005 Sep;139(5):439–57.
74. Bédard M, Molloy DW, Squire L, Dubois S, Lever JA, O'Donnell M. The Zarit Burden Interview. Gerontologist. 2001 Oct 1;41(5):652–7.

75. Yanny B, Pham N V., Saleh H, Saab S. Approaches to assessing Burden in caregivers of patients with cirrhosis. J Clin Transl Hepatol. 2020;8(2):127–34.
76. Bajaj JS, Wade JB, Gibson DP, Heuman DM, Thacker LR, Sterling RK, et al. The multi-dimensional burden of cirrhosis and hepatic encephalopathy on patients and caregivers. American Journal of Gastroenterology. 2011 Sep;106(9):1646–53.
77. Bédard M, William Molloy D, Squire L, Dubois S, Lever JA. The Zarit Burden Interview: A New Short Version and Screening Version [Internet]. Vol. 41, The Gerontologist. 2001. Available from: https://academic.oup.com/gerontologist/article/41/5/652/596578
78. Laetitia Y, Van Hooff RJ, De Smedt A, Alexis VE, Van Rita D, De Rohny VC, et al. Feasibility of ambulance-based telemedicine (fact) study: Safety, feasibility and reliability of third generation ambulance telemedicine. PLoS One. 2014 Oct 24;9(10).
79. Ramsey SD, Willke RJ, Glick H, Reed SD, Augustovski F, Jonsson B, et al. Cost-effectiveness analysis alongside clinical trials II - An ISPOR good research practices task force report. Value in Health. 2015 Mar 1;18(2):161–72.
80. Glick H. Analysis of Cost Data. 2022 Feb.
81. Ramdzan AR, Manaf MRA, Aizuddin AN, Latiff ZA, Teik KW, Ch'ng GS, et al. Cost-effectiveness of colorectal cancer genetic testing. Int J Environ Res Public Health. 2021 Aug 1;18(16).
82. Husereau D, Drummond M, Augustovski F, de Bekker-Grob E, Briggs AH, Carswell C, et al. Consolidated health economic evaluation reporting standards 2022 (CHEERS 2022) statement: updated reporting guidance for health economic evaluations. Int J Technol Assess Health Care. 2022 Jan 11;38(1).
83. Thiry N, Neyt M, Van De Sande S, Cleemput I. BELGIAN GUIDELINES FOR ECONOMIC EVALUATIONS: SECOND EDITION. Int J Technol Assess Health Care. 2014 Mar 30;30(6):601–7.
84. Siebert U, Alagoz O, Bayoumi AM, Jahn B, Owens DK, Cohen DJ, et al. State-transition modeling: A report of the ISPOR-SMDM modeling good research practices task force-3. Medical Decision Making. 2012 Sep;32(5):690–700.
85. Moro D, Schlander M, Telser H, Sola-Morales O, Clark MD, Olaye A, et al. Evaluating discrete choice experiment willingness to pay [DCE-WTP] analysis and relative social willingness to pay [RS-WTP] analysis in a health technology assessment of a treatment for an ultra-rare childhood disease [CLN2]. Expert Rev Pharmacoecon Outcomes Res. 2022;22(4):581–98.
86. Hauber AB, González JM, Groothuis-Oudshoorn CGM, Prior T, Marshall DA, Cunningham C, et al. Statistical Methods for the Analysis of Discrete Choice Experiments: A Report of the ISPOR Conjoint Analysis Good Research Practices Task Force. Value in Health. 2016 Jun 1;19(4):300–15.
87. Torri F, Schirinzi E, Fontanelli L, Ricci G, Mancuso M, Bochicchio M, et al. Telemedicine and remote monitoring in neuromuscular diseases: Challenges and opportunities. Available from: https://us.
88. Nipp RD, Gaufberg E, Vyas C, Azoba C, Qian CL, Jaggers J, et al. Full-length article available online at [Internet]. Vol. 2022, JCO Oncol Pract. Available from: https://doi.org/10.
89. Patel HY, West DJ. Hospital at Home: An Evolving Model for Comprehensive Healthcare. Global Journal on Quality and Safety in Healthcare. 2021 Nov 1;4(4):141–6.
90. Chua CMS, Ko SQ, Lai YF, Lim YW, Shorey S. Perceptions of Hospital-at-Home Among Stakeholders: a Meta-synthesis. Vol. 37, Journal of General Internal Medicine. Springer; 2022. p. 637–50.
91. Saenger P, Federman AD, DeCherrie L V., Lubetsky S, Catalan E, Leff B, et al. Choosing Inpatient vs Home Treatment: Why Patients Accept or Decline Hospital at Home. J Am Geriatr Soc. 2020 Jul 1;68(7):1579–83.
92. Kadakia KT, Balatbat CA, Siu AL, Cohen IG, Wilkins CH, Dzau VJ, et al. Hospital-at-Home: Multistakeholder Considerations for Program Dissemination and Scale. Vol. 100, Milbank Quarterly. John Wiley and Sons Inc; 2022. p. 673–701.
93. Levine DM, Pian J, Mahendrakumar K, Patel A, Saenz A, Schnipper JL. Hospital-Level Care at Home for Acutely Ill Adults: a Qualitative Evaluation of a Randomized Controlled Trial. J Gen Intern Med. 2021 Jul 1;36(7):1965–73.
94. DeCherrie L V., Wajnberg A, Soones T, Escobar C, Catalan E, Lubetsky S, et al. Hospital at Home-Plus: A Platform of Facility-Based Care. Vol. 67, Journal of the American Geriatrics Society. Blackwell Publishing Inc.; 2019. p. 596–602.

95. Wilson A, Wynn A, Parker H. Patient and carer satisfaction with “Hospital at Home”: quantitative and qualitative results from a randomised controlled trial. British Journal of General Practice. 2002.
96. Ko SQ, Chua CMS, Koh SH, Lim YW, Shorey S. Experiences of Patients and Their Caregivers Admitted to a Hospital-at-Home Program in Singapore: a Descriptive Qualitative Study. J Gen Intern Med. 2023 Feb 1;38(3):691–8.
97. Cai S, Intrator O, Chan C, Buxbaum L, Haggerty MA, Phibbs CS, et al. Association of Costs and Days at Home with Transfer Hospital in Home. JAMA Netw Open. 2021 Jun 29;4(6).
98. Zhao X, Sun X, Jing J, Zhou H, Jin Y. Safety study of Folfox-HAIC in relieving bed restriction. Journal of Interventional Medicine. 2021 Nov 1;4(4):203–7.
99. Dos Santos RC, Melo GAA, Silva RA, da Silva FLB, Viana Júnior AB, Caetano JÁ. Relationship between the comfort level of chronic renal patients and sociodemographic and clinical variables. Rev Bras Enferm. 2020;73.
100. Melo GAA, Aguiar LL, Silva RA, Quirino G da S, Pinheiro AKB, Caetano JÁ. Factors related to impaired comfort in chronic kidney disease patients on hemodialysis. Rev Bras Enferm. 2019;72(4):889–95.
