# Supplementary material for: Intrathecal pump refills at home or at the hospital: Protocol for a randomized controlled crossover trial—The IMPROVE study
Source: PLoS One. 2026 Jul 27;21(7):e0354092. doi: 10.1371/journal.pone.0354092 (PMC13405089; doi:10.1371/journal.pone.0354092)

Supporting information

Intrathecal pump refills at home or at the hospital: protocol for a randomized controlled crossover trial – the IMPROVE study

Ulrike Van Hoey[1¶*], Britt Winnepenninckx[1¶*], Maarten Moens[1,2,3,4,5,7&], Koen Putman[6], Lisa Goudman[1,2,3,4,5&]

S2 Figure. Intrathecal pump refill protocol. Step-by-step protocol of all procedures involved in intrathecal pump refills, including patient positioning, pump readout, preparation of prescribed medication, skin disinfection, aseptic access to the pump reservoir, intrathecal pump refill, and needle removal.

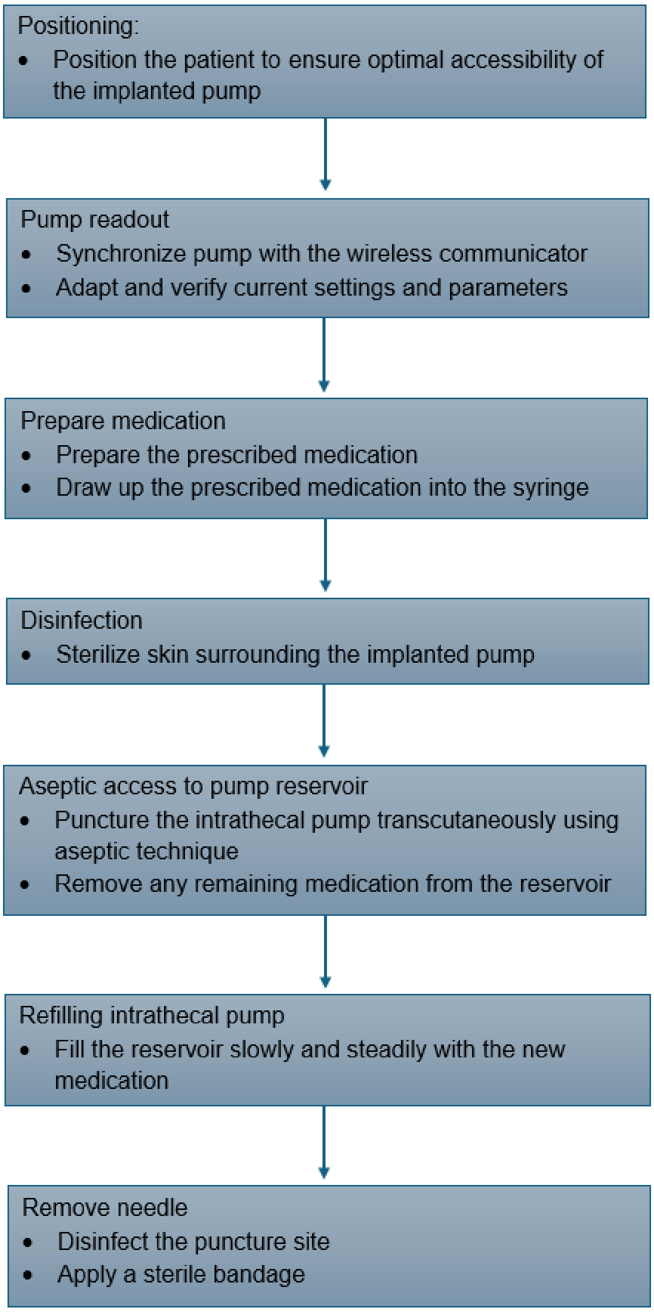

Supplement: S2 Fig — Step-by-step protocol of all procedures involved in intrathecal pump refills, including patient positioning, pump readout, preparation of prescribed medication, skin disinfection, aseptic access to the pump reservoir, intrathecal pump refill, and needle removal. (PDF) [file pone.0354092.s004.pdf]
