# Supplementary material for: Intrathecal pump refills at home or at the hospital: Protocol for a randomized controlled crossover trial—The IMPROVE study
Source: PLoS One. 2026 Jul 27;21(7):e0354092. doi: 10.1371/journal.pone.0354092 (PMC13405089; doi:10.1371/journal.pone.0354092)

Supporting information

Intrathecal pump refills at home or at the hospital: protocol for a randomized controlled crossover trial – the IMPROVE study

Ulrike Van Hoey[1¶*], Britt Winnepenninckx[1¶*], Maarten Moens[1,2,3,4,5,7&], Koen Putman[6], Lisa Goudman[1,2,3,4,5&]

S3 Figure. Differences between hospital- and home-based pump refills. Comparison of the procedures according to the refill setting, type of supervision, verification process, and follow-up.

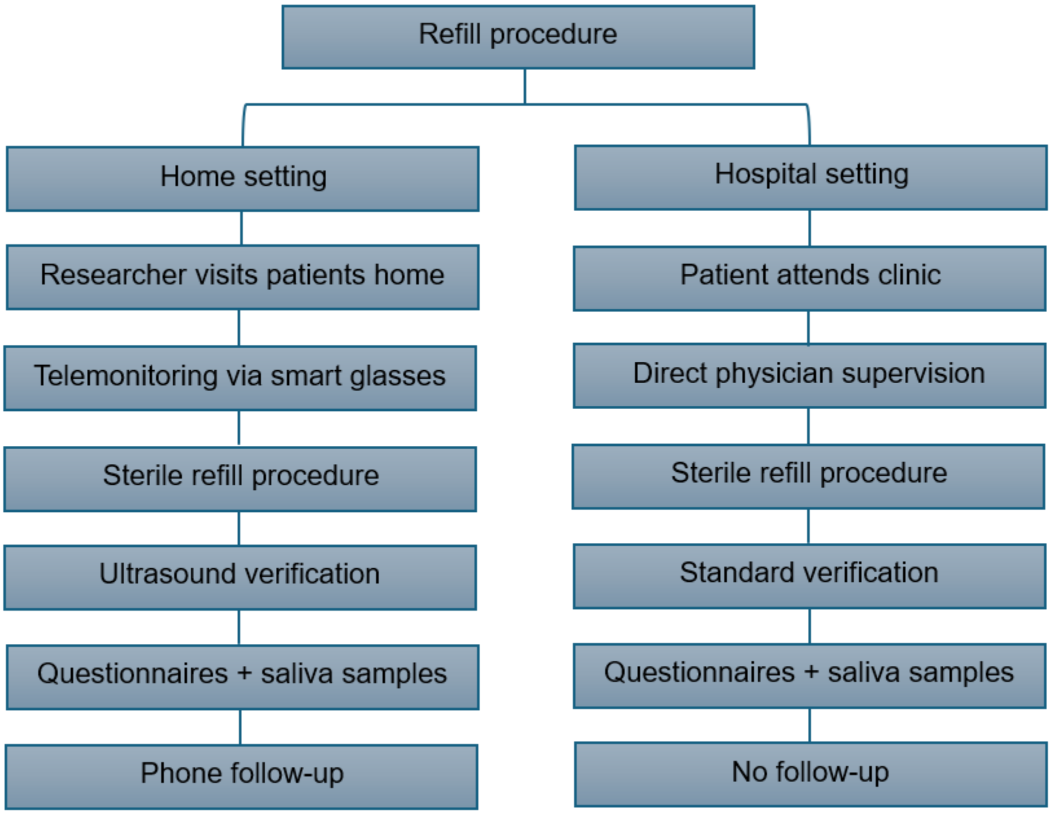

Supplement: S3 Fig — Comparison of the procedures according to the refill setting, type of supervision, verification process, and follow-up. (PDF) [file pone.0354092.s005.pdf]
