# Supplementary material for: Intrathecal pump refills at home or at the hospital: Protocol for a randomized controlled crossover trial—The IMPROVE study
Source: PLoS One. 2026 Jul 27;21(7):e0354092. doi: 10.1371/journal.pone.0354092 (PMC13405089; doi:10.1371/journal.pone.0354092)
Supplement: S5 Fig — Checklist covering pre-visit preparation, required materials, home environment setup, the refill procedure, post-procedure care, and follow-up. (PDF) [file pone.0354092.s007.pdf]

Supporting information

Intrathecal pump refills at home or at the hospital: protocol for a randomized controlled crossover trial – the IMPROVE study

Ulrike Van Hoey[1¶*], Britt Winnepenninckx[1¶*], Maarten Moens[1,2,3,4,5,7&], Koen Putman[6], Lisa Goudman[1,2,3,4,5&]

S5 Figure. Implementation checklist for home-based intrathecal pump refills. Checklist covering pre-visit preparation, required materials, home environment setup, the refill procedure, post-procedure care, and follow-up.

Home-based intrathecal pump refill: implementation checklist

Pre-visit preparation

- ✓ Verify medication prescription and dosage
- ✓ Confirm appointment and patient availability

Required materials

- ✓ Sterile refill kit
- ✓ Prescribed intrathecal medication
- ✓ Clinician programmer and wireless communicator
- ✓ Telemonitoring device
- ✓ Ultrasound device

Home environment setup

- ✓ Clean working surface
- ✓ Patient positioned comfortably and safely
- ✓ Minimization of distractions and interruptions

Procedure

- ✓ Refill according to protocol

Post procedure

- ✓ Ultrasound confirmation
- ✓ Scheduling next refill

Follow-up

- ✓ Telephone follow-up within defined timeframe (2–12 hours after refill)
- ✓ Adverse event reporting if applicable
