# Supplementary material for: Intrathecal pump refills at home or at the hospital: Protocol for a randomized controlled crossover trial—The IMPROVE study
Source: PLoS One. 2026 Jul 27;21(7):e0354092. doi: 10.1371/journal.pone.0354092 (PMC13405089; doi:10.1371/journal.pone.0354092)
Supplement: S1 Table — The expected outcomes between home and hospital refill settings are compared. (PDF) [file pone.0354092.s008.pdf]

Supporting information

Intrathecal pump refills at home or at the hospital: protocol for a randomized controlled crossover trial – the IMPROVE study

Ulrike Van Hoey[1¶*], Britt Winnepenninckx[1¶*], Maarten Moens[1,2,3,4,5,7&], Koen Putman[6], Lisa Goudman[1,2,3,4,5&]

S1 Table. Expected clinical outcomes of home versus hospital refills. Hypothesized differences in the reported outcomes (comfort, health-related quality of life, pain intensity, pain interference, stress, anxiety, self-efficacy, caregiver burden, safety, health expenditure and patient preference) between refill settings.

Outcome	Home-based refill	Hospital-based refill
Comfort	Higher perceived comfort due to familiar environment, and reduced travel burden.	Standard clinical environment; may provide increased sense of safety due to immediate access to medical resources, but associated with travel burden and hospital-related stress.
Health-related quality of life	Potential improvement due to reduced travel burden and increased convenience.	Standard care experience; travel required may reduce convenience.
Pain intensity and pain interference	Pain intensity expected to be comparable to hospital-based procedure, although perceived pain may be influenced by reduced pre-procedural stress and absence of travel-related burden.	Standard clinical procedure; pain perception may be influenced by hospital environment, travel-related stress, and waiting times.
Stress	Cortisol levels may be reduced due to lower anticipatory stress related to travel and the hospital environment. However, home-based care may also elicit context-specific stress, depending on individual patient perceptions of receiving medical treatment at home.	Cortisol levels may be higher due to hospital-associated anticipatory stress, travel burden, and clinical environment. However, the hospital setting may also provide reassurance in terms of immediate medical availability.
Anxiety	May be associated with lower anxiety due to reduced travel burden, familiar environment, and increased sense of control. However, some patients may experience increased anxiety	May be associated with higher anticipatory anxiety due to hospital environment, travel, and waiting times. However, some patients may experience reduced anxiety due to perceived safety and

	related to receiving medical procedures in the home setting.	immediate access to medical support.
Self-efficacy	May be associated with higher self-efficacy due to increased exposure to care in the home setting, greater patient involvement, and enhanced sense of autonomy and control over treatment management.	May be associated with lower self-efficacy due to more passive role in treatment delivery. However, some patients may experience increased confidence due to perceived professional support in a clinical environment.
Caregiver burden	May reduce caregiver burden by eliminating transportation needs and reducing logistical demands associated with hospital visits. However, increased caregiver involvement in the home setting may also contribute to perceived responsibility and emotional burden.	May increase logistical burden related to transportation and scheduling, although the clinical setting may reduce perceived caregiver responsibility during the procedure.
Safety	Potential risk of delayed response in complications. Potentially lower exposure to hospital pathogens (depending on home environment).	Immediate access to full hospital resources. Standard infection control in clinical setting.
Health expenditure	This may lower patient-related and indirect healthcare costs by reducing the need for transportation and limiting hospital visits. However, it can also introduce additional expenses, such as staff travel, portable equipment, and the organization of home visits.	Centralized care delivery can simplify logistics for healthcare providers, but it may lead to increased patient transportation costs and greater use of hospital resources.
Patient preference	May be preferred due to increased convenience, reduced travel burden, familiar surroundings, and perceived autonomy. However, some patients may prefer to separate medical care from the home environment.	It may be favoured because of its perceived safety, familiarity with the clinical environment, and immediate access to healthcare resources, despite the greater logistical burden.
